# Interrogating the *Plasmodium* Sporozoite Surface: Identification of Surface-Exposed Proteins and Demonstration of Glycosylation on CSP and TRAP by Mass Spectrometry-Based Proteomics

**DOI:** 10.1371/journal.ppat.1005606

**Published:** 2016-04-29

**Authors:** Kristian E. Swearingen, Scott E. Lindner, Lirong Shi, Melanie J. Shears, Anke Harupa, Christine S. Hopp, Ashley M. Vaughan, Timothy A. Springer, Robert L. Moritz, Stefan H. I. Kappe, Photini Sinnis

**Affiliations:** 1 Institute for Systems Biology, Seattle, Washington, United States of America; 2 Center for Infectious Disease Research, formerly Seattle Biomedical Research Institute, Seattle, Washington, United States of America; 3 Center for Malaria Research, Pennsylvania State University, University Park, Pennsylvania, United States of America; 4 Johns Hopkins Malaria Research Institute and Department of Molecular Microbiology and Immunology, Johns Hopkins Bloomberg School of Public Health, Johns Hopkins University, Baltimore, Maryland, United States of America; 5 Harvard Medical School, Boston, Massachusetts, United States of America; The Scripps Research Institute, UNITED STATES

## Abstract

Malaria parasite infection is initiated by the mosquito-transmitted sporozoite stage, a highly motile invasive cell that targets hepatocytes in the liver for infection. A promising approach to developing a malaria vaccine is the use of proteins located on the sporozoite surface as antigens to elicit humoral immune responses that prevent the establishment of infection. Very little of the *P*. *falciparum* genome has been considered as potential vaccine targets, and candidate vaccines have been almost exclusively based on single antigens, generating the need for novel target identification. The most advanced malaria vaccine to date, RTS,S, a subunit vaccine consisting of a portion of the major surface protein circumsporozoite protein (CSP), conferred limited protection in Phase III trials, falling short of community-established vaccine efficacy goals. In striking contrast to the limited protection seen in current vaccine trials, sterilizing immunity can be achieved by immunization with radiation-attenuated sporozoites, suggesting that more potent protection may be achievable with a multivalent protein vaccine. Here, we provide the most comprehensive analysis to date of proteins located on the surface of or secreted by *Plasmodium falciparum* salivary gland sporozoites. We used chemical labeling to isolate surface-exposed proteins on sporozoites and identified these proteins by mass spectrometry. We validated several of these targets and also provide evidence that components of the inner membrane complex are in fact surface-exposed and accessible to antibodies in live sporozoites. Finally, our mass spectrometry data provide the first direct evidence that the *Plasmodium* surface proteins CSP and TRAP are glycosylated in sporozoites, a finding that could impact the selection of vaccine antigens.

## Author Summary

Malaria remains one of the most important infectious diseases in the world, responsible for an estimated 500 million new cases and 600,000 deaths annually. The etiologic agents of the disease are protozoan parasites of the genus *Plasmodium* that have a complex cycle between mosquito and mammalian hosts. Though all clinical symptoms are attributable to the blood stages, it is only by attacking the transmission stages that we can make an impact on the economic and health burdens of malaria. Infection is initiated when mosquitoes inoculate sporozoites into the skin as they probe for blood. Sporozoites must locate blood vessels and enter the circulation to reach the liver where they invade and grow in hepatocytes. The inoculum is low and these early stages of infection are asymptomatic. Though the small amounts of material available for study has made large scale -omics studies difficult, killing the parasite at this stage would prevent infection and block downstream transmission to mosquitoes, thus preventing spread of disease. Here we use state-of-the-art biochemistry tools to identify the proteins on the sporozoite surface and find that two of the most studied proteins, CSP and TRAP, have post-translational modifications. These studies will aid investigations into the novel biology of sporozoites and importantly, significantly expand the pool of potential vaccine candidates.

## Introduction

Malaria remains one of the major global infectious diseases, responsible for nearly 438,000 deaths and 150 to 300 million new infections annually (World Malaria Report 2015, WHO). This disease, found in much of the tropical and subtropical regions of the world, is perpetuated through the mosquito-borne transmission of a eukaryotic parasite of the genus *Plasmodium*. Malaria infection is initiated when an infected anopheline mosquito takes a blood meal, and by doing so deposits the sporozoite form of the parasite into the skin. Sporozoites are motile and leave the bite site by traversing into the vasculature, traveling through the blood stream and ultimately invading hepatocytes in the liver. Liver stage parasites develop for approximately one week and release exo-erythrocytic merozoites into the blood stream to begin the blood stage of infection (reviewed in [1–2]). During the blood stage of infection, the iterative cycles of replication lead to high parasite numbers and to all clinical symptoms of malaria. Targeting the asymptomatic sporozoite and liver stage parasites, a time when parasite numbers are low, can lead to elimination of the parasite before it advances to the symptomatic stage of disease.

Malaria remains such a formidable disease in part due to the lack of effective approved vaccines and the ability of the parasite to rapidly evolve drug resistance [3–4]. Major strides have been made with RTS,S, the first malaria vaccine candidate to show efficacy in Phase III clinical trials. This has produced great enthusiasm for eventually meeting the goal of eradication. However, the efficacy and longevity metrics of RTS,S still fall below the community established efficacy goals [5], providing approximately 50% efficacy in preventing infection and 47% efficacy in protection from severe disease. [6–7]. Follow-up studies with RTS,S and other vaccine approaches on immunized volunteers indicated that antibody titers specific for sporozoites correlate with protection [8–10], suggesting that targeting the extracellular sporozoite stage may be an effective approach. One obvious shortcoming of the RTS,S vaccine is that it is composed of a single protein found on the sporozoite surface, the circumsporozoite protein (CSP). Conversely, powerful sterilizing immunity can be achieved by immunization with radiation-attenuated sporozoites. A recent study using protein arrays to probe the antibody repertoire of individuals immunized with irradiated sporozoites found 77 parasite proteins were associated with sterile protection against sporozoites [11], suggesting that a multivalent anti-sporozoite vaccine targeting several surface-exposed antigens will likely induce more potent protection.

There is a wealth of studies focused upon identifying additional sporozoite antigens that could ultimately be part of a multivalent subunit vaccine in addition to CSP (reviewed in [12]). Sporozoite antigens currently being assessed in Phase I and Phase II clinical trials include apical membrane antigen 1 (AMA1), liver stage antigen 1 (LSA1), circumsporozoite-related antigen (Exp-1), and thrombospondin-related anonymous protein/sporozoite surface protein 2 (TRAP) [13] (see the WHO Rainbow Table [14]). Several of these antigens, when formulated as recombinant proteins with different adjuvants, or when expressed from a viral vector, have conferred protection by inducing antibody and T-cell responses. However, the selection of an optimal antigen cocktail would be greatly advanced by having an extensive, experimentally validated list of surface proteins. Several efforts have been made to identify the repertoire of sporozoite proteins [15–17]. Our recent work has resulted in the most comprehensive sporozoite proteome to date, as well as a preliminary study of the sporozoite surface proteome in which 14 putative surface proteins were identified [18].

In this study, we identify novel putative surface proteins of *Plasmodium falciparum* salivary gland sporozoites, and confirm that key targets remain surface-exposed in response to treatment with molecular mimics of the host environments that the sporozoite encounters. We additionally provide evidence that components of the inner membrane complex (IMC) are in fact surface-exposed and accessible to antibodies, thus opening this protein group up for consideration in vaccine target selection. Finally, we provide evidence that two leading vaccine candidates, CSP and TRAP, are glycosylated in their thrombospondin type 1 repeat (TSR) domains. Understanding such protein modifications is crucial in the design of effective antibody-based vaccines.

## Results

### Identification of Surface-Exposed Proteins

We used chemical labeling and mass spectrometry-based proteomics to identify putatively surface-exposed proteins of *P*. *falciparum* salivary gland sporozoites. Sporozoites were obtained by dissection of salivary glands from infected mosquitoes and then purified twice on an Accudenz gradient as previously described [19]. Live parasites were treated with a cell-impermeable, amine-reactive tag [20] that attached a biotin moiety to surface-exposed lysine residues and N-termini. Subsequently, parasites were lysed and biotin-labeled proteins were purified using streptavidin affixed to magnetic beads. The affinity-purified proteins were eluted and fractionated by SDS-PAGE. Peptides resulting from in-gel digestion with trypsin were analyzed by nanoLC-MS/MS employing an LTQ Velos Pro-Orbitrap Elite. Mass spectrometry data were analyzed with the Trans-Proteomic Pipeline [21]. The data presented here only includes proteins identified with a ProteinProphet probability corresponding to a false discovery rate (FDR) less than one percent. A total of 349 *Plasmodium* proteins were identified from six biological replicates of surface-labeled salivary gland sporozoites (S2 Table). A total of 50 proteins were identified from three biological replicates of unlabeled controls, of which 47 were also found in the labeled samples (S3 Table). The six labeled replicates consisted of two sub-groups representing two different laboratories collecting and preparing the samples. A total of 51 proteins were identified from the group 1 samples, of which 49 were among the 347 proteins identified from the group 2 samples. The laboratory preparing samples, the amount of starting material, and endogenous and exogenous contamination (e.g. residual mosquito protein, streptavidin and BSA from magnetic dynabeads) had a large effect on protein recovery, and the number of parasite proteins identified varied widely, ranging from 27 to 313. Despite this variability, all of the proteins identified from the group 1 samples as highly likely to be surface-exposed on sporozoites were also identified as high-quality candidates from the group 2 samples.

As was observed from a similar analysis of the ookinete surface proteome [22], we identified many intracellular components that are unlikely to be present at the surface of the sporozoite, e.g., histones and ribosomal proteins. While control experiments with unlabeled sporozoites revealed that non-specific binding of high-abundance intracellular proteins was not entirely prevented in our experiments, the abundance of the proteins identified from controls was quite low, in contrast to the relatively high abundance of proteins identified from labeled sporozoites (S7 Table). It is more likely that the majority of these intracellular proteins originated from parasites with compromised plasma membranes. Indeed, when we examined the permeability of purified sporozoites prior to biotinylation using propidium iodide, a cell-impermeant dye that enters dead or dying sporozoites, we found that approximately 10% of sporozoites were permeable to the dye. Thus, it is not altogether surprising that highly-abundant intracellular proteins would be biotinylated and found in our dataset. Despite this confounding factor, many of the proteins with the strongest evidence for being enriched by the method employed were validated as truly surface-exposed on sporozoites, as we show below.

In order to identify high-quality surface antigens, we used experimental and theoretical information to devise a prioritization scheme. We first identified those proteins with the strongest evidence of having been truly enriched by the biotinylation strategy. We employed label-free quantification methods to assess enrichment relative to unlabeled controls, using the program SAINT [23] as well as comparing the spectral abundance factor (SAF) [24] by t-test. These two quantitative methods agreed well; SAINT identified as significantly-enriched the same 110 proteins identified by t-test, plus an additional 46 (S2 Table). For some high-abundance proteins (e.g. CSP), addition of the biotin tag to a lysine residue could be directly detected in a portion of the identifying mass spectra, providing direct evidence that the protein was labeled (S6 Table). Failure to detect labeled peptides for a protein does not mean that the protein was not labeled [25], so we did not consider absence of this evidence as evidence against enrichment. It was then necessary to distinguish truly surface-exposed proteins from intracellular proteins that were likely labeled due to compromised plasma membranes in a small number of sporozoites, as we discuss above. Accordingly, we used established tools using a protein’s primary sequence to predict the presence of surface protein characteristics, i.e., a signal sequence, transmembrane domain or glycosylphosphatidylinositol (GPI) anchor addition sequence.

We identified 42 proteins that are highly likely to be present on the surface of salivary gland sporozoites (Table 1). These proteins, the strongest candidates identified by our prioritization scheme, are known or predicted to have surface protein characteristics and were significantly enriched by the biotinylation strategy. The seven proteins with direct evidence for biotinylation were designated Tier 1 candidates and the other 35 were designated Tier 2. An additional 58 proteins, designated Tier 3, had predicted surface protein characteristics but were not significantly enriched compared to unlabeled controls (S2 Table). Although proteins in this category had weaker experimental evidence for enrichment, they may still be viable targets for future studies. Indeed, included in this category are several known surface proteins, e.g., gamete egress and sporozoite traversal protein (GEST; PF3D7_1449000) and sporozoite protein essential for cell traversal (SPECT1; PF3D7_1342500). Priority tiers 4 and 5 are comprised of 114 proteins that do not have predicted surface characteristics but were significantly enriched, some with spectral evidence for labeling (Tier 4). The majority of these are likely intracellular proteins that were labeled due to compromised plasma membranes, as discussed above. However, as we will discuss below, some of these proteins which are thought to be primarily intercellular may in fact be surface-exposed on sporozoites.

**Table 1:** Putative surface-exposed proteins in salivary gland sporozoites.

Accession Number	Description	Observed (of 6 replicates)	Cellular Function	Labeled^a^	SP/TM/GPI^b^
PF3D7_0304600	circumsporozoite (CS) protein (CSP)	6	Invasion & Migration	YES	Signal, GPI
PF3D7_0818600	BEM46-like protein, putative (PBLP)	6	Invasion & Migration	YES	TM
PF3D7_0104000	thrombospondin-related sporozoite protein (TRSP)	6	Invasion & Migration	YES	Signal, TM
PF3D7_1335900	thrombospondin-related anonymous protein (TRAP)	6	Invasion & Migration	YES	Signal
PF3D7_0919500	sugar transporter, putative	6	Transporter	YES	12 TMs
PF3D7_1222300	endoplasmin, putative (GRP94)	6	Chaperone	YES	Signal
PF3D7_0511400	conserved Plasmodium protein, unknown function	4	Hypothetical	YES	Signal, TM
PF3D7_0812300	sporozoite surface protein 3, putative (SSP3)	6	Invasion & Migration	NO	Signal, TM
PF3D7_0204700	hexose transporter (HT)	6	Transporter	NO	12 TMs
PF3D7_1446900	glutaminyl-peptide cyclotransferase, putative	6	Metabolism	NO	TM
PF3D7_1324900	L-lactate dehydrogenase (LDH)	6	Metabolism	NO	TM
PF3D7_1133400	apical membrane antigen 1 (AMA1)	6	Invasion & Migration	NO	Signal, TM
PF3D7_0408600	sporozoite invasion-associated protein 1 (SIAP1)	5	Invasion & Migration	NO	Signal
PF3D7_0508000	6-Cys protein (P38)	5	Invasion & Migration	NO	Signal, GPI
PF3D7_0620000	conserved Plasmodium protein, unknown function	4	Hypothetical	NO	Signal, GPI
PF3D7_0918000	glideosome-associated protein 50 (GAP50)	4	Motility	NO	Signal, GPI
PF3D7_0917900	heat shock protein 70 (HSP70-2)	4	Chaperone	NO	Signal
PF3D7_1406800	glideosome associated protein with multiple membrane spans 3 (GAPM3)	4	Motility	NO	6 TMs
PF3D7_1028900	inner membrane complex protein 1m, putative (IMC1m)	3	Cytoskeleton	NO	TM
PF3D7_1311800	M1-family alanyl aminopeptidase (M1AAP)	3	Protease	NO	TM
PF3D7_0828800	GPI-anchored micronemal antigen (GAMA)	3	Invasion & Migration	NO	Signal, GPI
PF3D7_0827900	protein disulfide isomerase (PDI8)	3	Chaperone	NO	Signal
PF3D7_1430700	NADP-specific glutamate dehydrogenase (GDH2)	3	Metabolism	NO	Signal
PF3D7_1411100.1/2	conserved Plasmodium membrane protein, unknown function	3	Hypothetical	NO	8 TMs
PF3D7_0506900	rhomboid protease ROM4 (ROM4)	3	Protease	NO	6 TMs
PF3D7_0423500	glideosome associated protein with multiple membrane spans 2 (GAPM2)	3	Motility	NO	6 TMs
PF3D7_1323700	glideosome associated protein with multiple membrane spans 1 (GAPM1)	3	Motility	NO	6 TMs
PF3D7_1237700	conserved protein, unknown function	3	Hypothetical	NO	5 TMs
PF3D7_1238000	COPI associated protein, putative	3	Vesicular Trafficking	NO	4 TMs
PF3D7_1011500	conserved Plasmodium membrane protein, unknown function	3	Hypothetical	NO	4 TMs
PF3D7_1409400	conserved Plasmodium membrane protein, unknown function	3	Hypothetical	NO	4 TMs
PF3D7_0316700	HVA22/TB2/DP1 family protein, putative	3	Hypothetical	NO	3 TMs
PF3D7_1037300	ADP/ATP transporter on adenylate translocase (ADT)	3	Transporter	NO	3 TMs
PF3D7_0408700	sporozoite micronemal protein essential for cell traversal (PLP1)	3	Invasion & Migration	NO	2 TMs
PF3D7_0515700	glideosome-associated protein 40, putative (GAP40)	3	Motility	NO	10 TMs
PF3D7_1326000	conserved Plasmodium protein, unknown function	2	Hypothetical	NO	TM
PF3D7_1252100	rhoptry neck protein 3 (RON3)	2	Invasion & Migration	NO	Signal, 3 TMs
PF3D7_1457000	signal peptide peptidase (SPP)	2	Signaling	NO	8 TMs
PF3D7_0508200	longevity-assurance (LAG1) protein, putative	2	Stress Response	NO	7 TMs
PF3D7_1132800	aquaglyceroporin (AQP)	2	Transporter	NO	6 TMs, Signal
PF3D7_0316600	formate-nitrite transporter (FNT)	2	Transporter	NO	6 TMs
PF3D7_1146300	conserved Plasmodium protein, unknown function	2	Hypothetical	NO	2 TMs

^a^Evidence for incorporation of the biotin label was found in the identifying mass spectra.

^b^ SP, signal peptide; TM, transmembrane domain; GPI, glycosylphosphatidylinositol anchor

**Fig 1:**
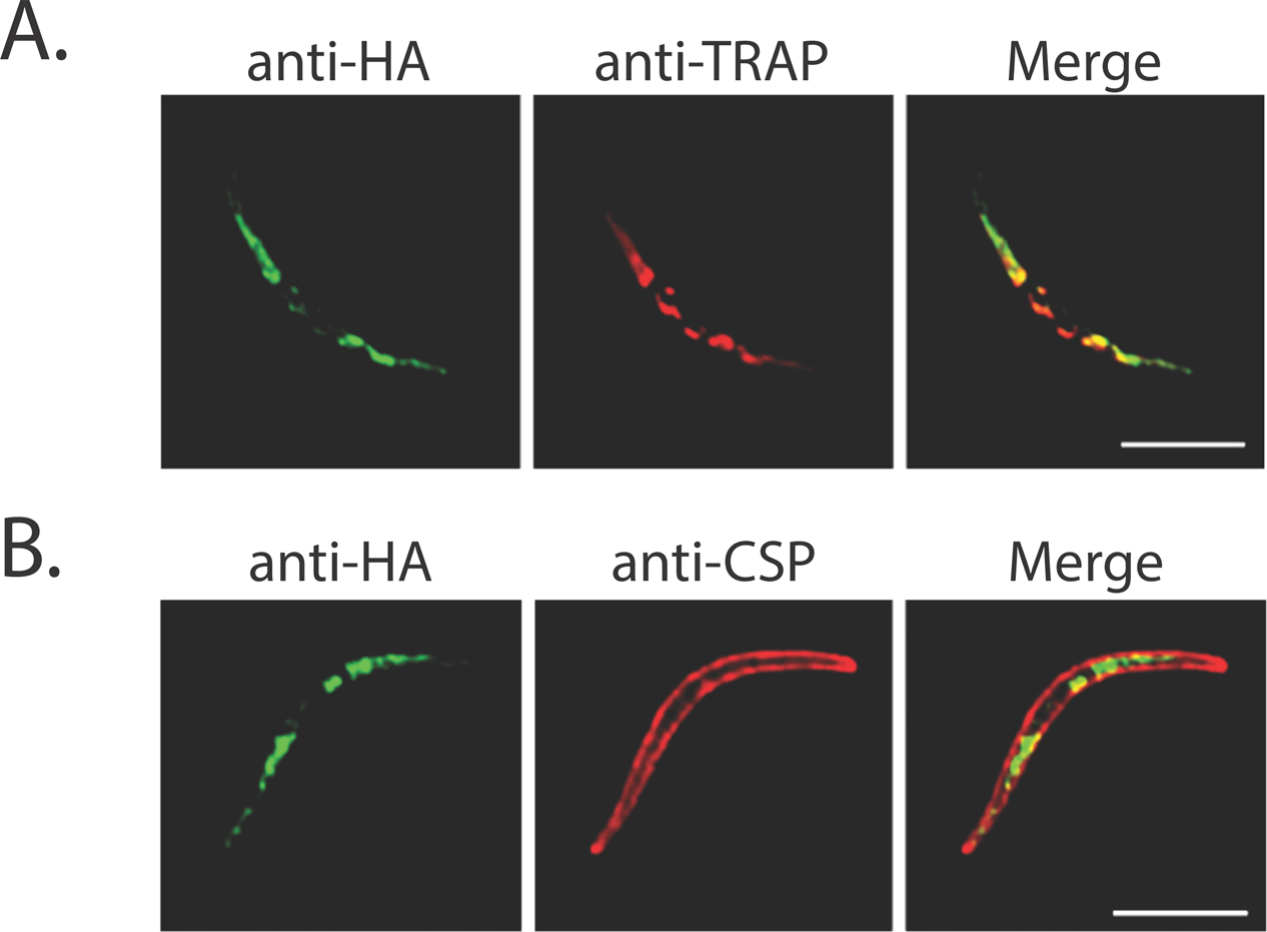
The 6-Cys protein p38 is a microneme protein. Transgenic salivary gland sporozoites expressing p38 with an HA tag were subjected to an indirect fluorescence assay (IFA). p38 was localized using antibodies against the HA tag (green) and parasites were co-stained with antibodies specific for TRAP (A) or CSP (B). Scale bar is 5 microns.

Not surprisingly, CSP (PF3D7_0304600) was among the strongest of the tier 1 candidates, identified in every biological replicate of labeled sporozoites as the most abundant *Plasmodium* protein and exhibiting strong evidence for incorporation of the biotin label. Similarly strong evidence (in terms of enrichment, abundance and labeling) was found for thrombospondin-related sporozoite protein (TRSP; PF3D7_0104000) and the putative BEM46-like protein PBLP (PF3D7_0818600). Interestingly, TRAP (PF3D7_1335900), a known micronemal protein that is translocated to the sporozoite surface during motility, was not nearly so well-enriched by our biotinylation method. Both TRAP and CSP were among the 10 most abundant proteins in our previously-published sporozoite proteome and exhibited nearly identical abundance [18] (S7 Table), but TRAP was over 30-fold less abundant than CSP in this study of surface proteins. This data is consistent with the fact that, unlike CSP, the majority of TRAP is intracellular in salivary gland sporozoites [26] (Fig 1) and suggests that some TRAP secretion was likely induced over the course of sample handling.

We assigned each of the proteins in Table 1 to functional categories based on the PlasmoDB annotation (Version 26; [27]) as well as their published function in sporozoites or erythrocytic stages when known. If a protein’s function was not known, functional class was assigned based on the literature on other organisms. As would be expected, proteins involved in sporozoite motility, migration through tissue, and invasion were well-represented. Unexpectedly, some chaperone and metabolic proteins were also identified. While these may be experimental artifacts, there is increasing evidence that proteins from these classes are found on the surface of both prokaryotic and eukaryotic pathogens where they function as virulence factors [28–29]. The three chaperone proteins in Table 1, endoplasmin (PF3D7_1222300), HSP70-2 (PF3D7_0917900) and PDI8 (PF3D7_0827900), are known to localize to the endoplasmic reticulum. All three contain predicted signal sequences at their N-termini in addition to endoplasmic reticulum retention sequences at their C-termini [30–32]. Interestingly, homologs of HSP70-2 and endoplasmin (grp78/BiP and gp96, respectively) can be found on the surface of certain cell types in vertebrates [33–34]. Further, endoplasmin was identified as a putatively surface-exposed protein in *P*. *berghei* ookinetes [22]. Other work with *Plasmodium* ookinetes demonstrated that they express enolase (PF3D7_1015900), GAPDH (PF3D7_1462800) and even actin (PF3D7_1246200) [35–37] on their surface, and that these proteins function during migration through the mosquito midgut [35–36]. Further, recent work demonstrated a critical role for the chaperone HSP20 (PF3D7_0816500) in gliding motility [38]. Enolase, GAPDH, actin and HSP20 were among the Tier 4 and 5 proteins in our data set, identified as significantly-enriched compared to unlabeled controls but lacking predicted characteristics of surface proteins (S2 Table). Taken together, these studies provide support for the notion that the metabolic enzymes and chaperones we identified in our study may have important moonlighting functions in the sporozoite.

### Activating Sporozoites Reveals Additional Surface-Exposed Proteins

The invasive stages of apicomplexan parasites have specialized apical organelles termed micronemes and rhoptries whose regulated secretion is required for active migration and ultimately for host cell invasion. This has been best demonstrated with *Toxoplasma gondii* and *Plasmodium* merozoites where material is not limiting [39–41]. Sporozoites possess apical organelles and express many of the same microneme and rhoptry proteins found in merozoites. In contrast to merozoites, however, sporozoites have a significant migration phase prior to host cell invasion, and though some apical organellar proteins overlap, others are likely to be unique. In order to determine how the sporozoite surface changes as sporozoites migrate through different environments, such as the skin and hepatic sinusoids, we performed a proteomic analysis of surface-exposed proteins in sporozoites treated with compounds previously reported to be associated with sporozoite migration and invasion. Incubation with bovine serum albumin (BSA) mimics arrival in the mammalian host and induces gliding motility [42]. Incubation with heparin induces proteolytic cleavage of the major surface protein, CSP, and mimics arrival at the liver, initiating a switch from migration to cell invasion [43–44]. We analyzed three BSA-treated replicates (S4 Table) and three heparin-treated replicates (S5 Table) and applied the same criteria for labeling and enrichment as were applied to the untreated dataset (Table 2). There was large overlap in the proteins identified from untreated and treated sporozoites, though a few proteins were only identified or enriched from treated sporozoites, notably the 6-Cys protein P12p. Importantly, the highest-confidence proteins identified from untreated sporozoites (i.e., those identified in nearly every replicate and significantly enriched compared to controls) were also identified as likely surface proteins in the treated sporozoites. Interestingly, there was more spectral evidence for labeling in the treated sporozoites, especially from BSA treatment (S6 Table). These data suggest the possibility that certain sporozoite surface proteins were more accessible to label upon exposure to chemicals that mimic environments encountered in the mammalian host.

**Table 2:** Putative surface-exposed proteins identified from BSA- and heparin-treated salivary gland sporozoites.

Accession Number	Description	BSA			Heparin			Untreated		
		Obs.^a^	Enriched^b^	Label^c^	Obs.^a^	Enriched^b^	Label^c^	Obs.^a^	Enriched^b^	Label^c^
PF3D7_0304600	circumsporozoite (CS) protein (CSP)	3	YES	YES	3	YES	YES	6	YES	YES
PF3D7_0818600	BEM46-like protein, putative (PBLP)	3	YES	YES	3	YES	YES	6	YES	YES
PF3D7_0104000	thrombospondin-related sporozoite protein (TRSP)	3	YES	YES	3	YES	YES	6	YES	YES
PF3D7_1446900	glutaminyl-peptide cyclotransferase, putative	3	YES	YES	3	YES	YES	6	YES	-
PF3D7_0408600	sporozoite invasion-associated protein 1 (SIAP1)	3	YES	YES	2	-	YES	5	YES	-
PF3D7_0812300	sporozoite surface protein 3, putative (SSP3)	3	YES	YES	3	YES	-	6	YES	-
PF3D7_1133400	apical membrane antigen 1 (AMA1)	3	YES	YES	3	YES	-	6	YES	-
PF3D7_0919500	sugar transporter, putative	3	YES	YES	3	YES	-	6	YES	YES
PF3D7_0508000	6-Cys protein (P38)	3	YES	YES	3	YES	-	5	YES	-
PF3D7_0612800	6-Cys protein (P12p)	2	YES	YES	1	-	-	1	-	-
PF3D7_0104100	conserved Plasmodium membrane protein, unknown function	2	YES	YES	-	-	-	-	-	-
PF3D7_1449000	gamete egress and sporozoite traversal protein, putative (GEST)	3	-	YES	-	-	-	3	-	-
PF3D7_0620000	conserved Plasmodium protein, unknown function	3	YES	-	3	YES	-	4	YES	-
PF3D7_1011500	conserved Plasmodium membrane protein, unknown function	3	YES	-	3	YES	-	3	YES	-
PF3D7_1335900	thrombospondin-related anonymous protein (TRAP)	3	YES	-	3	-	-	6	YES	YES
PF3D7_0204700	hexose transporter (HT)	3	YES	-	2	-	-	6	YES	-
PF3D7_1222300	endoplasmin, putative (GRP94)	3	YES	-	3	-	-	6	YES	YES
PF3D7_0506900	rhomboid protease ROM4 (ROM4)	3	YES	-	1	-	-	3	YES	-
PF3D7_1406800	glideosome associated protein with multiple membrane spans 3 (GAPM3)	3	YES	-	1	-	-	4	YES	-
PF3D7_0515700	glideosome-associated protein 40, putative (GAP40)	3	YES	-	1	-	-	3	YES	-
PF3D7_1324900	L-lactate dehydrogenase (LDH)	3	YES	-	-	-	-	6	YES	-
PF3D7_1452000	rhoptry neck protein 2 (RON2)	3	YES	-	-	-	-	3	-	-
PF3D7_0918000	glideosome-associated protein 50 (GAP50)	3	YES	-	-	-	-	4	YES	-
PF3D7_0917900	heat shock protein 70 (HSP70-2)	3	YES	-	-	-	-	4	YES	-
PF3D7_1431900	inner membrane complex suture component, putative (ISC3)	3	YES	-	-	-	-	1	-	-
PF3D7_0316600	formate-nitrite transporter (FNT)	2	YES	-	-	-	-	2	YES	-
PF3D7_0408700	sporozoite micronemal protein essential for cell traversal (PLP1)	2	YES	-	-	-	-	3	YES	-
PF3D7_0704600	E3 ubiquitin-protein ligase (UT)	-	-	-	1	-	YES	3	-	-

^a^Number of biological replicates in which the protein was observed, out of three total (BSA- or heparin-treated) or six total (untreated).

^b^Protein was significantly more abundant in labeled samples compared to unlabeled controls.

^c^Evidence for incorporation of the biotin label was found in the identifying mass spectra.

### Validation of Surface Exposure of a Sugar Transporter and the 6-Cys Protein p38

In order to provide additional evidence for our list of proteins that are putatively surface-exposed on salivary gland sporozoites, we tagged two whose localization has not been previously investigated. We used the rodent malaria parasite *Plasmodium yoelii* for these experiments as this species is more amenable to genetic modification than is *P*. *falciparum*. Importantly, proteins thus far characterized in both *P*. *falciparum* and rodent malaria sporozoites have not differed in their subcellular localization (apiloc.biochem.unimelb.edu.au/apiloc/apiloc; [45]). We tagged the endogenous gene for a putative sugar transporter, PY17X_0823700 (ortholog of Pf3D7_0919500), and a 6-Cys protein, p38, PY17X_1108700 (ortholog of Pf3D7_0508000) with a triple HA tag on the C-terminus. Transgenic parasites were produced by single-crossover recombination for p38 or double-crossover recombination for the sugar transporter, and their correct integration was confirmed by genotyping PCR (S1 Fig and S8 Table). Transgenic parasites were fed to *An*. *stephensi* mosquitoes and salivary gland sporozoites were isolated and subjected to an indirect immunofluorescence assay (IFA).

**Fig 2:**
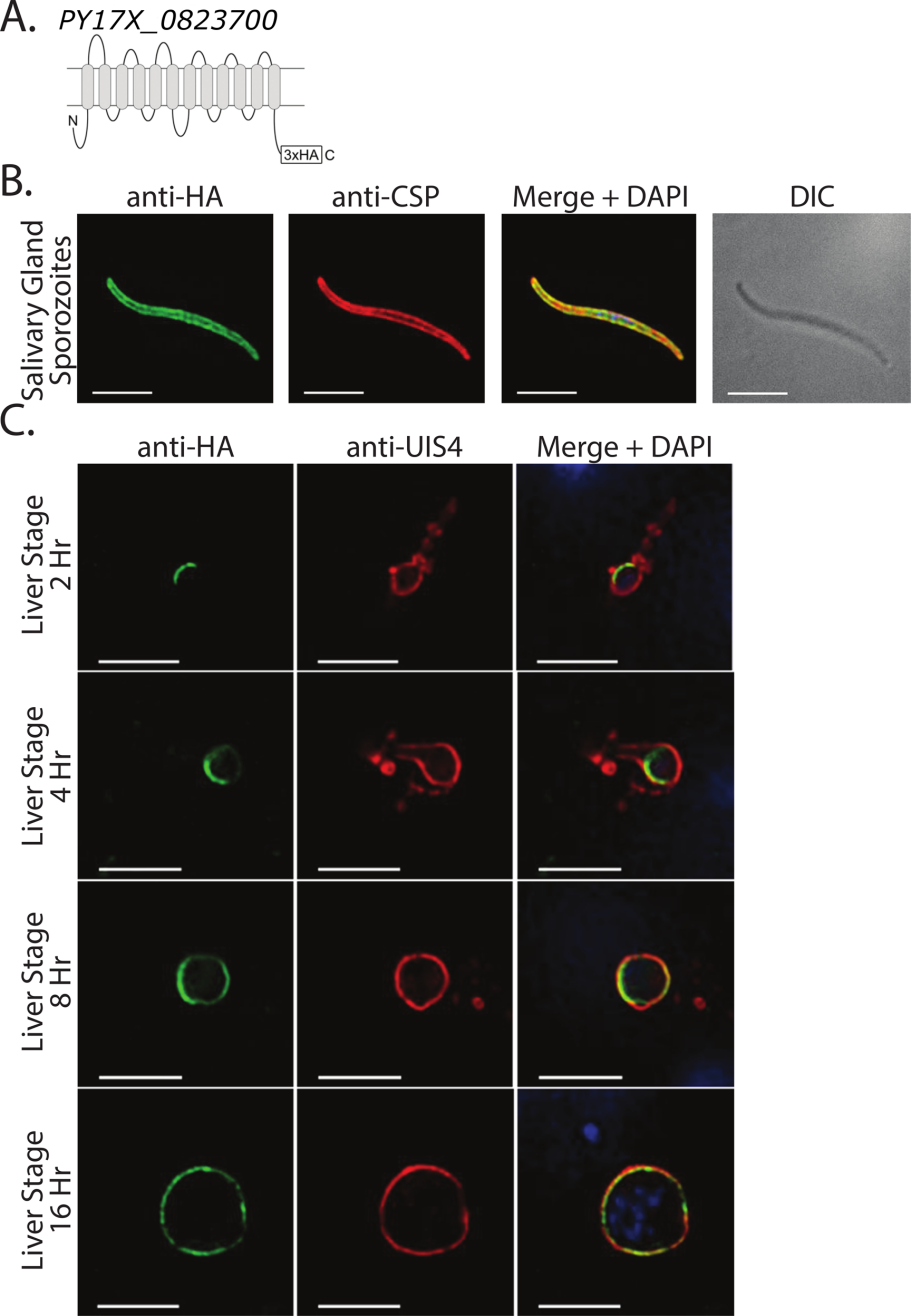
Localization of the sugar transporter in sporozoites and exoerythrocytic stages. (A) Schematic of the 12 transmembrane domains predicted to exist in the putative sugar transporter PY17X_0823700. Both N- and C-termini are predicted to be intracellular. An HA tag is appended to the C-terminus. (B) Transgenic salivary gland sporozoites were stained with antibodies against the HA tag (green) and co-stained with antibodies directed against the repeat region of CSP (red). Nucleic acids were labeled with DAPI. The differential interference contrast (DIC) image demonstrated overall sporozoite morphology. Scale bar is 5 microns. (C) Localization of sugar transporter in liver stage parasites at 2, 4, 8, and 16 h post-infection. Anti-HA staining (green) was adjacent and internal to anti-UIS4 staining (red). Scale bar is 5 microns.

The staining and localization of the 6-Cys protein p38 was internal to CSP and matched that of TRAP, a known micronemal protein that is secreted during motility and translocated to the sporozoite surface (Fig 1). In contrast, the staining and localization of the sugar transporter co-localized well with CSP, suggesting surface localization (Fig 2B). We also investigated localization of the sugar transporter during liver stage infection, co-staining with antibodies to the parasitophorous vacuole membrane marker UIS4 (Fig 2C). We observed the localization of the sugar transporter on the parasite plasma membrane located just interior to the PVM. This is most evident at 8 and 16 h post-infection. Further work is needed to determine the function(s) of the sugar transporter and other transporters in our dataset. Interestingly, expression of the gene for this sugar transporter is significantly higher in sporozoites than in other parasite stages [46]. It is therefore possible that this transporter is required for importing nutrients while the parasite is in mosquito salivary glands waiting to be inoculated into the next host, a phase that can last days to weeks, or for importing nutrients after their inoculation into the mammalian host, a time when energy resources are needed to fuel migration to the liver. Taken together, the localization data of p38 and the sugar transporter lends credence to the methods and prioritization scheme we employ here for identifying surface-exposed proteins.

### Components of the Inner Membrane Complex Are Accessible to Antibodies

**Fig 3:**
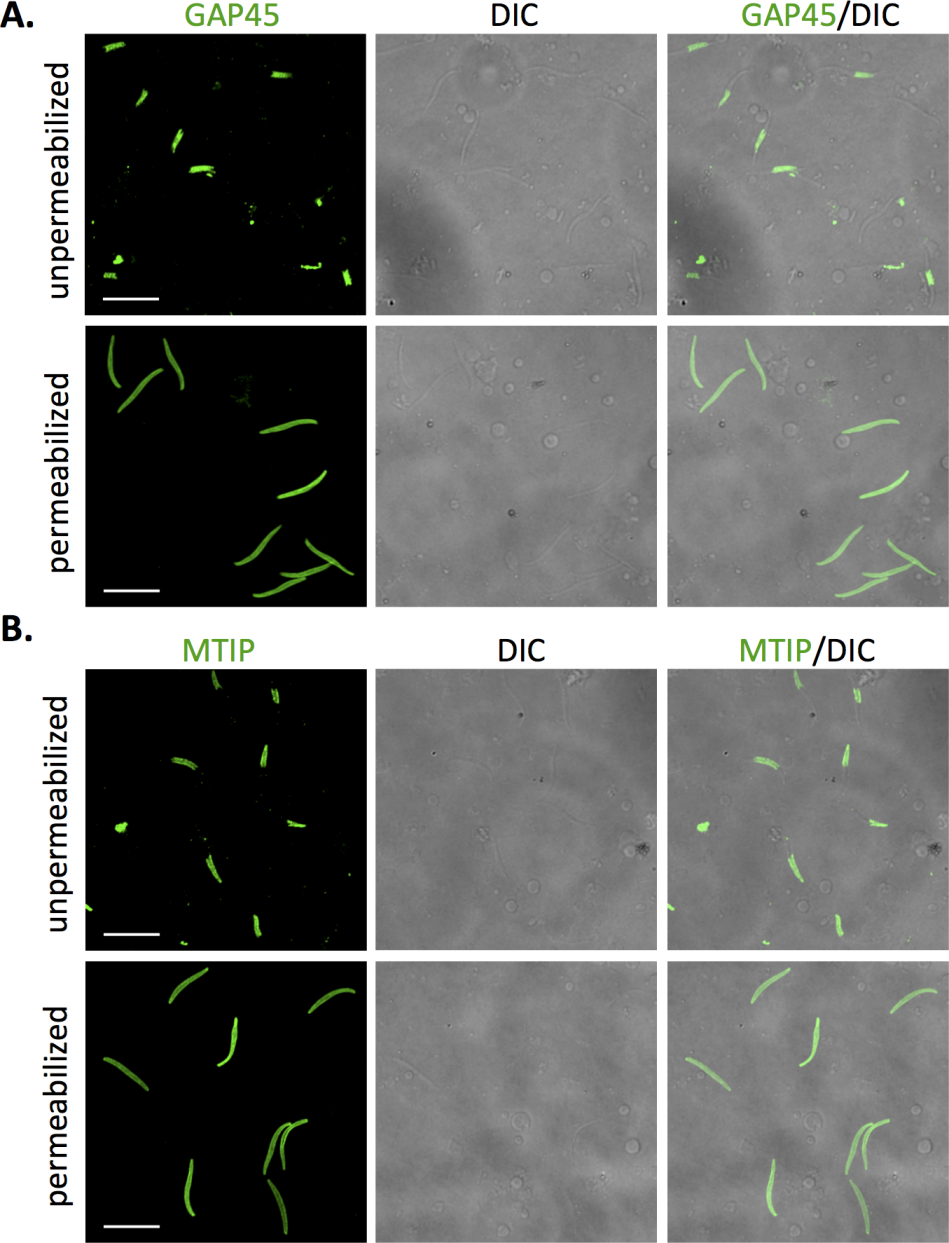
The inner membrane complex proteins MTIP and GAP45 are surface-exposed in unpermeabilized salivary gland sporozoites. Fluorescence microscopy of *P*. *falciparum* salivary gland sporozoites allowed to glide for 20 min and then stained for GAP45 (A) or MTIP (B). Sporozoites were fixed with 4% paraformaldehyde and either left unpermeabilized or permeabilized with Triton-X100. Shown are representative fluorescent images with their paired differential interference contrast (DIC) images. Unpermeabilized sporozoites featured strong patches of staining at the anterior or posterior ends, or in the middle of the sporozoite. In contrast, antibodies stained the entire sporozoite in permeabilized specimens. Scale bar is 10 microns.

Among the significantly-enriched proteins identified here were several components of the glideosome that are known to be located between the plasma membrane and the inner membrane complex (IMC), a double-membrane structure consisting of flattened cisternae located beneath the plasma membrane that plays a critical role in motility as well as interacting with the microtubule cytoskeleton and maintaining sporozoite structure [47–48]. Notably, the motor protein myosin A (MyoA, PF3D7_1342600) and its partner actin (ACT1, PF3D7_1246200) were identified in nearly every biological replicate of labeled sporozoites and exhibited spectral evidence for labeling. These proteins were not anticipated to be on the sporozoite surface. However, they were also identified in the only other surface proteome of a malaria parasite, the ookinete, which also exhibits gliding motility [22, 49]. To determine if this finding resulted from experimental artifact, or if in fact IMC proteins were truly exposed and more accessible to the external environment than previously considered, we performed IFAs on *P*. *falciparum* sporozoites that were spun onto coverslips and allowed to glide for 20 min, then paraformaldehyde-fixed. The sporozoites were then either permeabilized or not prior to staining with antibodies specific for the IMC proteins MTIP [50] and GAP45 [51] (Fig 3). Surprisingly, between 75 and 85% of the population of non-permeabilized sporozoites was stained by these antibodies, showing a restricted, banded pattern that was observed either at the anterior or posterior ends or in a patch in the middle section of the sporozoite. In contrast, permeabilized sporozoites were stained circumferentially in a pattern typical for IMC proteins [50] (Fig 3). We also performed MTIP and GAP45 staining of live *P*. *falciparum* sporozoites that had been allowed to glide for 20 min and then moved to the cold room and stained with the MTIP and GAP45 antibodies prior to fixation. By this methodology, a smaller proportion of total sporozoites stained with the antibodies, however, 38% and 13% of sporozoites reproducibly stained with MTIP or GAP45 antibodies, respectively (S2 Fig). To determine whether paraformaldehyde fixation led to permeabilization of sporozoites, we performed propidium iodide staining on live and fixed sporozoites. Only 5% of either live or fixed sporozoites incorporated the dye, suggesting that our fixation protocol did not significantly permeabilize the sporozoite. Taken together, these data indicate that either portions of the sporozoite plasma membrane are sufficiently permeable to allow antibody access to the IMC, or that some components of the motility machinery change localization during motility such that they become surface-exposed and accessible to the biotinylation reagent as well as to antibodies [52]. These data suggest that components of the IMC could be considered in the selection of antigen candidates.

### Glycosylation of *Plasmodium* Surface Proteins

**Fig 4:**
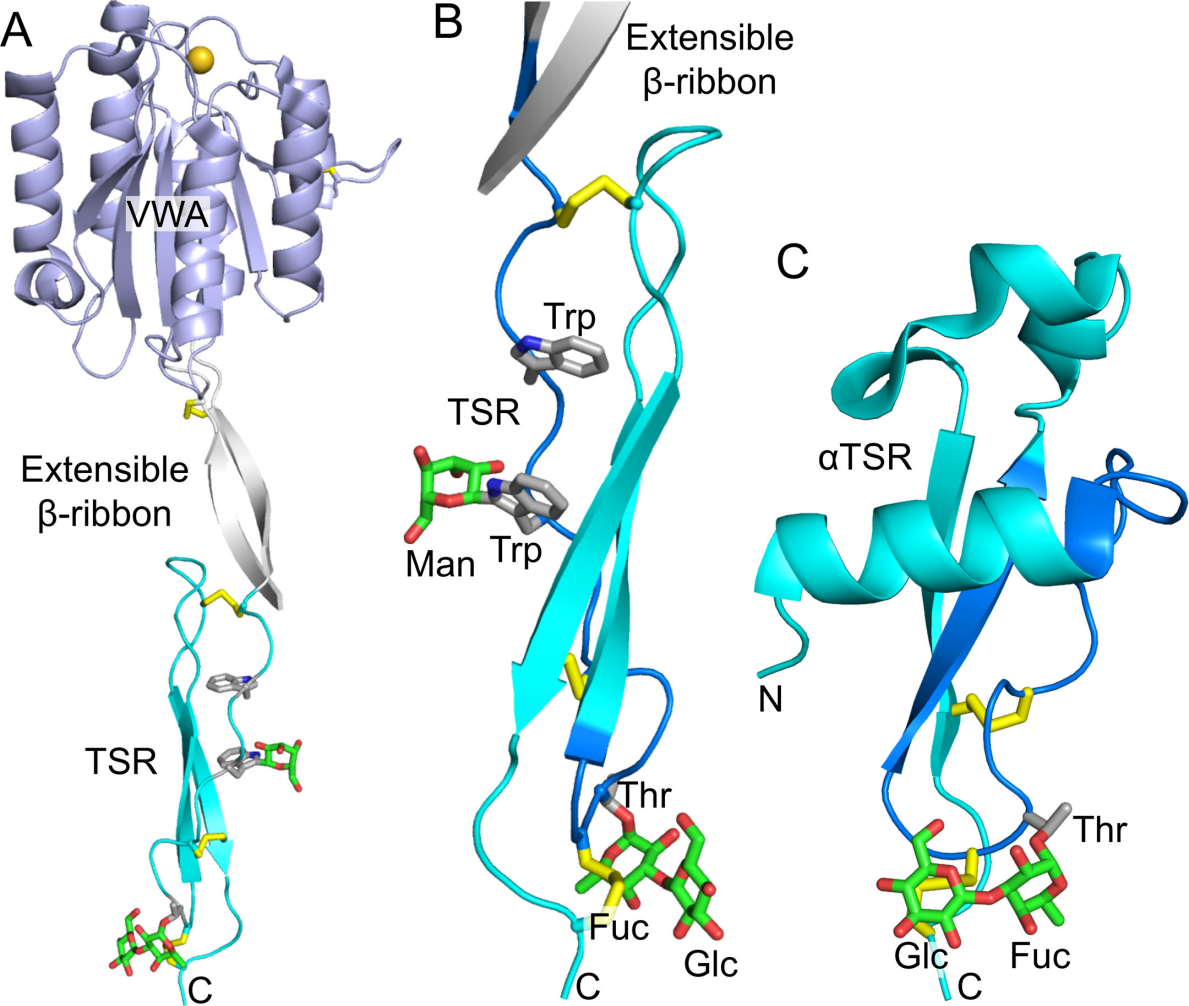
Ribbon diagrams of TRAP and CSP. (A & B) Three-dimensional models of TRAP extracellular domains (A) and detail of its TSR domain (B) showing the location of the observed glycosylation modifications. View in B is rotated ~180° about an axis vertical in the page relative to (A). TRAP from *P*. *vivax*, which is highly homologous to TRAP from *P*. *falciparum*, is shown, with the open, putative high-affinity state of the Von Willebrand factor type A (VWA) domain with its bound Mg^2+^ ion as a gold sphere [60]. (C) A three-dimensional model of the TSR domain of CSP showing location of the glycosylation event observed by mass spectrometry [61]. For all panels, sticks show glycans where carbons are green and oxygens are red. Amino acid side chains to which they are attached are shown with silver carbons and blue nitrogens. Disulfide bonds are shown in yellow. Side chains are shown for both Trp in the WXXW motif. TSR ribbons are in cyan, and in B and C, peptide segments identified by mass spectrometry with glycans attached are colored in marine. C-termini are marked with “C”. Mannose attached to Trp is modeled from the TRAP homologue in *Toxoplasma gondii* [62]; and the disaccharide attached to Thr in CSP is modeled from that in TRAP. Carbohydrate was added after superposition on the Trp or Thr side chain. The Trp and Thr residues have essentially identical orientations (rotamers) with and without the glycan. In both the TRAP structure and the CSP model, the glucose residue in the disaccharide has a similar role in burying the adjacent disulfide bond.

Our mass spectrometric analysis of *Plasmodium* surface proteins provides, for the first time, direct evidence of glycosylation on CSP and TRAP in salivary gland sporozoites. Both proteins contain a thrombospondin type 1 repeat (TSR), a cell-adhesion domain found in many proteins that functions in cell-cell interactions and cell guidance [53]. TSRs in other organisms have been shown to be O-fucosylated and C-mannosylated [54–55]. The motif CX_2-3_(S/T)CXXG in TSR domains can be modified with an O-linked fucose at the Ser/Thr [55], and this fucose can be further modified with glucose to produce a β1,3-linked disaccharide [56–57]. Additionally, the WXXW and WXXC motifs of TSR domains can be modified with a C-linked mannose at Trp [58–59]. These potential glycosylation motifs are present in the TSR domains of both CSP and TRAP in all *Plasmodium* species. X-ray crystallography and mass spectrometry studies on proteins expressed in mammalian cells showed that *Pf*CSP and *Pf*TRAP were O-fucosylated and that the TRAP homologue MIC2 in *T*. *gondii* was C-mannosylated in the predicted fashion in recombinant systems [60–62]. We now present direct evidence that CSP is modified by O-fucosylation and that TRAP is modified by both O-fucosylation and C-mannosylation in *P*. *falciparum* sporozoites (Fig 4).

While it is possible to identify peptide modifications using peptide spectrum matching search engines, this approach could not be used to search our data for O-fucosylation. O-linked glycans are highly labile in the gas phase and virtually all of the glycan is lost at the collision energies employed for peptide fragmentation, resulting in unmodified peptide fragments [63–64]. C-linked hexose is more stable, but collisional activation may lead to cross-ring cleavage of the glycan, resulting in a mass defect in the fragment spectra [58]. In order to determine if CSP and TRAP are glycosylated in salivary gland sporozoites, we manually investigated our nanoLC-MS/MS data for the presence of precursor peptide ions with masses corresponding to modification of the peptides in question. Because precursor ion spectra were collected in an Orbitrap, it was possible to match predicted and observed precursor ion masses with less than 2 ppm mass error. The identity of these peptide ions were then confirmed by manual annotation of the low-resolution fragmentation mass spectra collected in the ion trap.

**Fig 5:**
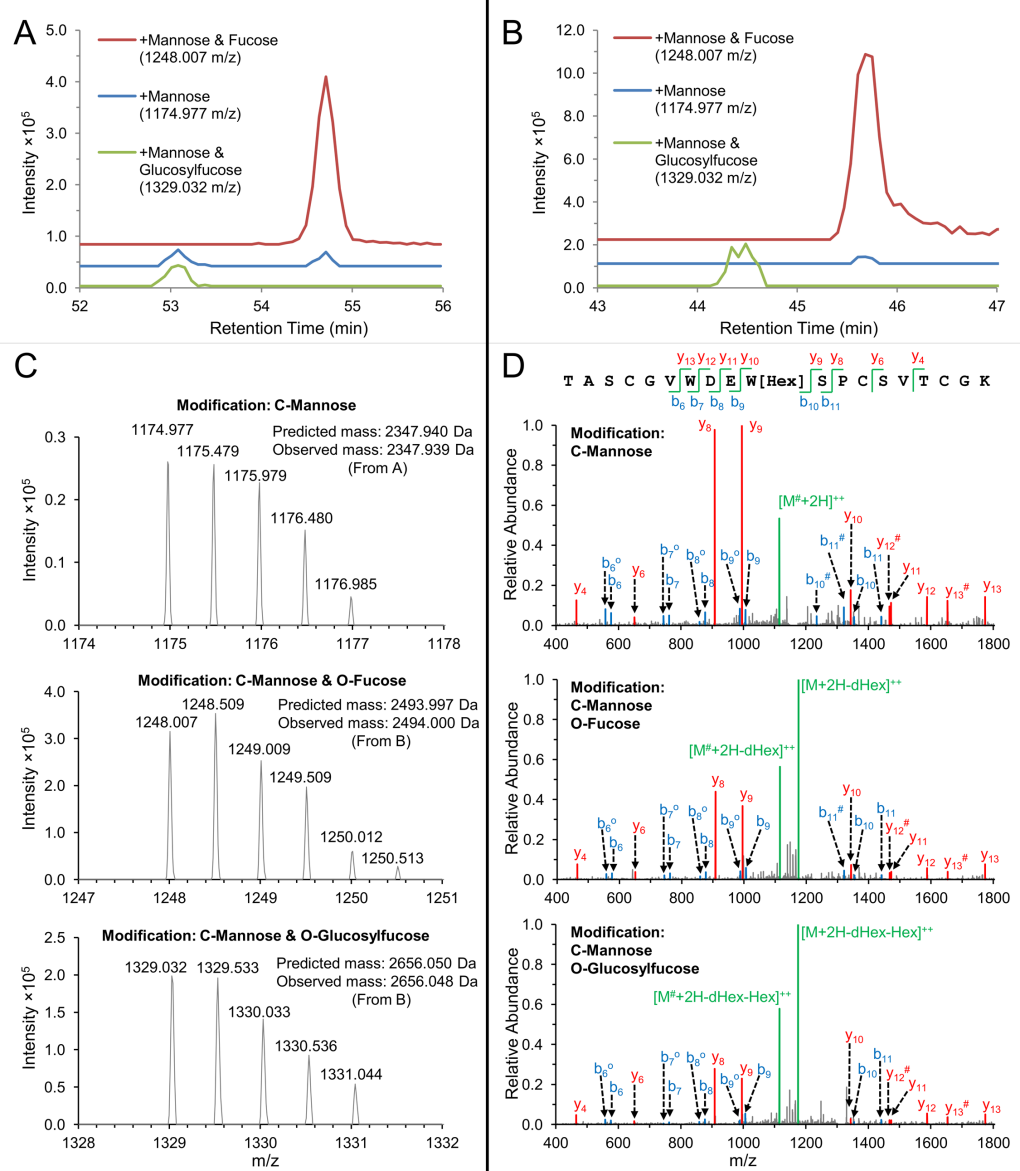
Mass spectral evidence for glycosylation of TRAP. (A & B) Representative extracted ion chromatograms (XIC) of the doubly-charged ions of the glycosylated TRAP peptide TASCGVWDEWSPCSVTCGK from *P*. *falciparum* salivary gland sporozoites. (A) XIC from an un-enriched sporozoite sample (data acquired from our previous work [18]). (B) XIC from a surface-labeled sporozoite sample (S1 Table). Traces are offset on the Y-axis for clarity. The mass-to-charge ratio (*m/z*) for each species is indicated. Representative precursor spectra from the species in (A) and (B) are shown in (C). The Orbitrap enabled measurement of peptide masses with high accuracy (<1 ppm mass error). The largest chromatographic peak (red) was produced by a species with a mass matching the peptide plus a hexose and a deoxyhexose, which we presume to be C-mannose and O-fucose. The peptide was also observed with a mass equal to two hexoses and a deoxyhexose (green), which we presume to be a C-mannose and an O-linked glucosylfucose disaccharide. A species was also observed with a mass matching the peptide modified only with C-mannose (blue). However, the peaks associated with this species co-eluted with the more heavily-modified peptides, indicating that they likely arose from loss of the O-linked glycan due to in-source fragmentation. (D) Representative collision-induced dissociation (CID) fragmentation spectra of the three peptide species. CID of the C-mannosylated peptide lacking O-fucose was obtained from (A), providing confirmation of the assignments of the fragment spectra obtained from the O-fucosylated species seen in (B). Fragment ions are annotated as b-ions (blue) and y-ions (red). The unfragmented peptide is designated M (green), with addition of protons (H) and neutral loss of hexose (Hex) and deoxyhexose (dHex). Ions are singly-charged unless indicated doubly-charged (^++^). Neutral loss of water (-18 Da) is indicated with (^o^). Loss of C_4_H_8_O_4_ (-120 Da) due to cross-ring cleavage of the C-mannose is indicated with (^#^). Fragmentation spectra of the O-fucosylated peptides show that the dominant product of CID fragmentation was the intact peptide that had lost the O-linked glycan but retained the C-mannose. The same species with partial loss of the C-mannose due to cross-ring fragmentation was the second most abundant species. The dominant peptide fragments retained the C-mannose, allowing confident localization of this glycan at the Trp of WSPC (indicated as W[Hex]). No peptide fragment spectra were observed with the O-fucose glycans intact, but based on the presence of the O-fucosylation motif, we presume that the Thr of CSVTCG is modified with O-fucose.

The TRAP tryptic peptide TASCGVWDEWSPCSVTCGK, which contains both the O-fucosylation motif as well as two of the C-mannosylation motifs described above, was only identified in its glycosylated form. The TRAP peptide was confidently identified from two ion species with distinct chromatographic retention times, one with a mass matching the addition of one deoxyhexose and one hexose, and the other with a mass corresponding to addition of one deoxyhexose and two hexoses (Fig 5). Of note, fucose is a deoxyhexose and mannose and glucose are hexoses. The dominant species in the fragment spectra for both of the putatively O-fucosylated peptides matched the mass of the peptide with addition of a single hexose, suggesting the presence of a single C-mannose which was retained at the collision energy employed while the O-linked fucose monosaccharide or glucosylfucose disaccharide was lost. The second most abundant fragment ion in the fragment spectra matched the mass of the hexosylated peptide with a loss of 120 Da, consistent with cross-ring cleavage of C-mannose. Several abundant fragment ions confidently placed the C-mannose at the C-terminal tryptophan of the motif WDEWSPC. No precursor ions were observed to suggest that the peptide was present in unmodified form or O-fucosylated without the presence of C-mannose; in fact, the unmodified peptide was never identified in any of the data sets presented here or in our previous analysis of the sporozoite proteome [18]. These data, combined with chromatographic elution profiles, suggest that TRAP in salivary gland sporozoites is entirely or almost entirely modified with both C-mannose and O-fucose. We observed modification with either a single fucose or with a glucosylfucose disaccharide. Based on chromatographic peak area, it appears that the disaccharide is the more prevalent modification.

**Fig 6:**
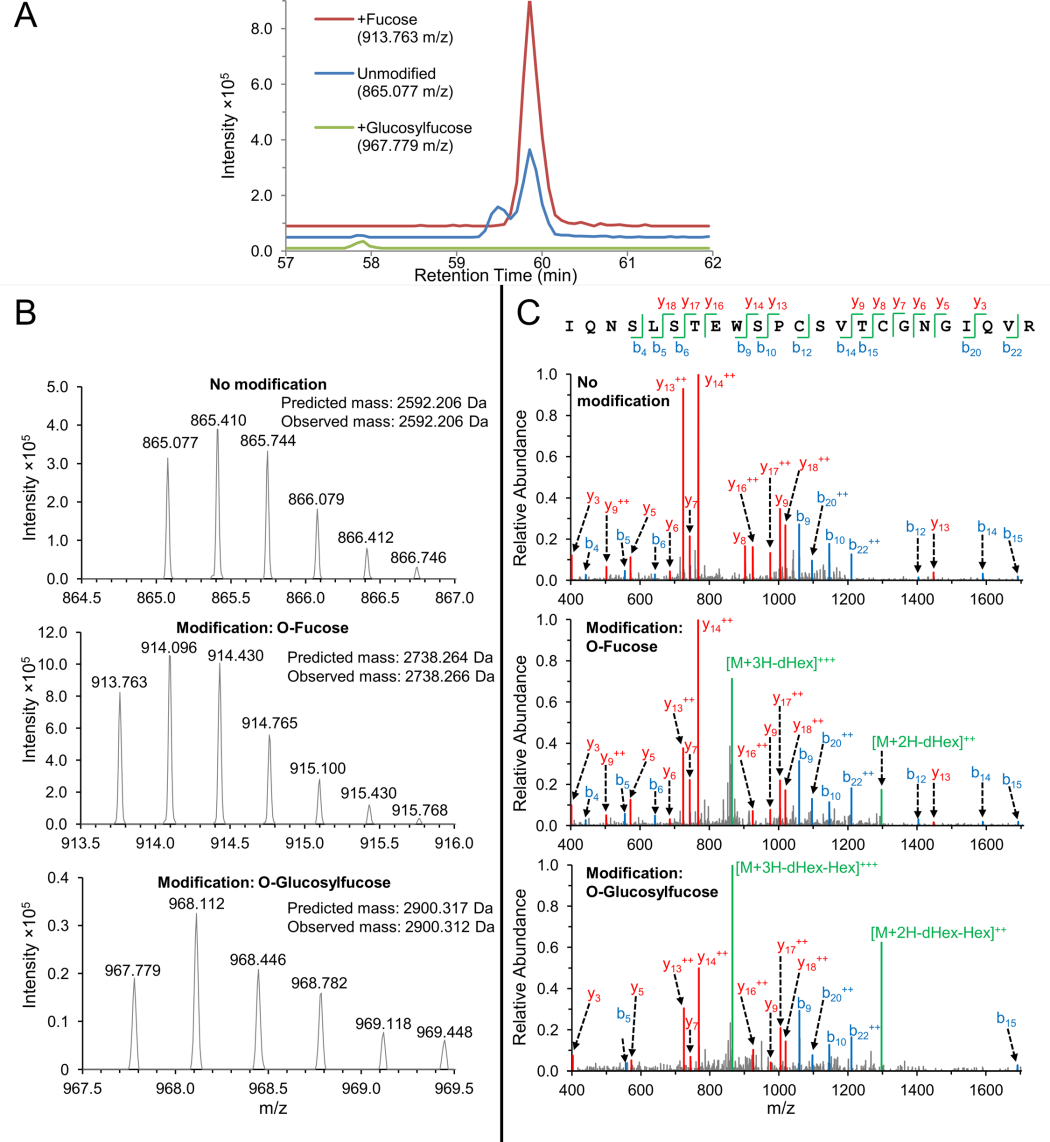
Mass spectral evidence for glycosylation of CSP. (A) Representative extracted ion chromatograms (XIC) of the triply-charged ions of the glycosylated CSP peptide IQNSLSTEWSPCSVTCGNGIQVR acquired from *P*. *falciparum* salivary gland sporozoites. (Data from an un-enriched sporozoite sample acquired in our previous work [18]). Traces are offset on the Y-axis for clarity. The mass-to-charge ratio (*m/z*) for each species is indicated. Representative precursor spectra from the species in (A) are shown in (B). The Orbitrap enabled measurement of peptide masses with high accuracy (<2 ppm mass error). The largest chromatographic peak (red) was produced by a species with a mass matching the peptide plus a deoxyhexose which we presume to be O-fucose. The peptide was also observed with a mass equal to a hexose and a deoxyhexose (green), which we presume to be an O-linked glucosylfucose disaccharide. Evidence for loss of O-linked glycans due to in-source fragmentation is seen as chromatographic peaks matching the unmodified peptide ion (blue) co-eluting with the O-fucosylated species. The unmodified peptide was also observed in a peak with distinct retention time, suggesting that the peptide was also present in the sample in unmodified form. (C) Representative collision-induced dissociation (CID) fragmentation spectra of the three peptide species are shown with annotated fragment b-ions (blue) and y-ions (red). The unfragmented peptide is designated M (green), with addition of protons (H) and neutral loss of hexose (Hex) and deoxyhexose (dHex). Ions are singly-charged unless indicated doubly-charged (^++^) or triply-charged (^+++^). Fragmentation spectra of the fucosylated peptides show that a primary product of CID fragmentation was the intact peptide that had lost the O-linked glycan. No peptide fragment spectra were observed with the O-fucose glycans intact, but based on the presence of the O-fucosylation motif, we presume that the Thr of CSVTCG is modified with O-fucose.

The CSP tryptic peptide IQNSLSTEWSPCSVTCGNGIQVR contains an O-fucosylation motif and a WXXC C-mannosylation motif, but unlike TRAP, the CSP peptide lacks the preceding WXXW motif. We identified this peptide both with and without modification (Fig 6). The modified peptide was confidently identified from precursor ions with masses corresponding to addition of a single deoxyhexose or one deoxyhexose and one hexose. Chromatographic peaks showed evidence that the unmodified peptide was also present (Fig 6). Unlike for TRAP, the fragment spectra of the putatively modified CSP peptides contained only unmodified fragment ions and exhibited no evidence of C-mannosylation. Furthermore, highly abundant fragment ions matched the mass of the intact, unmodified peptide precursor. These data suggest that in salivary gland sporozoites, most but not all CSP is modified with either O-fucose or an O-glucosylfucose disaccharide. Based on chromatographic peak area, it appears that the monosaccharide is the more prevalent modification.

## Discussion

In this study we have used chemical labeling and mass spectrometry to identify proteins on the surface of sporozoites, constitutively and after treatment with compounds that initiate gliding motility and invasion. We also present verification experiments on two of these identified proteins and provide evidence that some of the proteins of the sporozoite’s glideosome may be *bona fide* surface proteins, at least during a portion of the sporozoite’s life. Although the remainder of these proteins will require validation of their surface localization using orthogonal methodologies, this represents the largest dataset to date focused on the sporozoite surface and should fuel future research in the areas of sporozoite biology and pre-erythrocytic stage vaccine development. Importantly, our prioritized list of surface-expose antigens identifies candidates that should aid current vaccine efforts.

The list of putative sporozoite surface proteins we present here greatly expands on previous work from our group, which was preliminary and relied on a single biological replicate of salivary gland sporozoites [18]. In order to directly compare that dataset with our current data set, we reanalyzed the raw mass spectral data from that work using the databases and analysis parameters employed here (S9 Table). Excepting a putative GTPase, all *Plasmodium* proteins identified from our previous dataset were also identified here, the majority from multiple replicates and with more peptide spectrum matches than previously. Of these 27 proteins, 21 were significantly enriched in labeled sporozoites compared to unlabeled controls in this work, and 11 were among the high-priority proteins listed in Table 1, including CSP, TRAP, AMA1, TRSP and the sugar transporter. Importantly, this new work also identifies many putative surface proteins not identified in our preliminary work, and of these, we have validated p38 in this study.

The large overlap between the treatment sets (BSA and heparin) and the untreated protein dataset reflect our lack of knowledge about how sporozoites change their activation state, and related to this, our inability to synchronize sporozoites in any one state. Thus, the identification of known microneme proteins such as TRAP and TRSP in the untreated dataset is not surprising, as sporozoites isolated from salivary glands and put through further purification procedures are likely encountering signals that they have left the mosquito. On this background, the addition of BSA and heparin, which have observable effects on freshly dissected sporozoites, did not reveal as many differences as we expected to find in the complement of enriched proteins. However, these experiments with treated sporozoites did identify additional targets that were not enriched from the untreated samples, and several high-priority targets from the untreated samples were enriched as well or even better in conditions mimicking those found in the mammalian host. As our knowledge of sporozoite biology increases, these types of studies should yield more informative data on how the sporozoite’s surface changes as it migrates from mosquito midgut to mammalian liver.

Our lists of putative surface proteins include several that we did not expect to observe, such as chaperones and proteins that function in cellular metabolism. As stated earlier, a possible source of enriched cytosolic proteins is the small number of dead or distressed sporozoites that cannot be separated from the pool of sporozoites used for these analyses. However, some of these unexpected proteins may have moonlighting functions and actually spend some time on the sporozoite surface. Indeed, an increasing number of studies, with both bacterial and eukaryotic pathogens, suggest that this may be quite common [65–67].

Because the primary objective of this work was to identify proteins that are localized to the parasite surface, it was necessary to devise a means of prioritizing identified proteins for their likelihood of being truly surface-exposed. This was accomplished by combining predicted characteristics of membrane proteins with the experimental evidence for protein enrichment and incorporation of the biotin label. The ranking system we present here provides a scheme for rationally prioritizing the putative sporozoite surface proteins identified for future validation and vaccine development. Indeed, the top tier includes CSP and TRAP, components of vaccine candidates in Phase III and II clinical trials, respectively [7, 68], and we believe the other high-priority proteins are quality candidates for combining with CSP and TRAP in a multivalent subunit vaccine.

Until recently, it was not clear whether *Plasmodium* was capable of N- or O- glycosylation of proteins [69]. This lack of knowledge stemmed from the paucity of material available for study and contamination of that material with host proteins due to the obligate intracellular lifestyle of the parasite. Bioinformatic analyses demonstrate that the *Plasmodium* genome encodes only a few glycosyltransferases and thus, can synthesize only the truncated N-glycans, GlcNAc or (GlcNAc)_2_ [70]. Using lectins specific for these modifications, N-glycosylated proteins were found on intraerythrocytic stage parasites, though the identities of the proteins and their modifications has not been investigated [71]. To date, O-glycosylation has not been unequivocally demonstrated in *Plasmodium*, though the presence of specific sugar nucleotides and a protein O-fucosyltransferase in the genome indicate that O-glycosylation is possible [72]. Here we describe, for the first time, examples of O-fucosylated and C-mannosylated proteins in *Plasmodium*. These data are relevant to the malaria vaccine effort as the carbohydrate modifications we describe were found in CSP and TRAP, two of the leading pre-erythrocytic stage malaria vaccine candidates. Polysaccharides have long been known to be important antigenic determinants in immune responses to pathogens and must be considered when selecting targets for vaccine development. Their importance has been emphasized recently by broadly neutralizing anti-HIV gp120 antibodies that recognize combinatorial oligosaccharide/protein epitopes [73]. The O-fucosylation and C-mannosylation sites in both CSP and TRAP are highly conserved, and the same fucosylation and glucosylation of CSP and TRAP seen in sporozoites was also seen when these proteins were expressed in mammalian cells [60–61]. In contrast, mannosylation showed marked variation. TRAP has an orthologue in *T*. *gondii*, MIC2. The same WXXWXXC mannosylation motif is present in each, yet crystal structures showed that human cells mannosylated the first Trp in MIC2 [62] and neither Trp in TRAP [60, 62], while mass spectrometry here showed that the second Trp in TRAP was mannosylated in sporozoites. These differences emphasize the importance of chemical characterization of parasite post-translational modifications in vaccine design.

The enzymes necessary for these modifications are expressed in *Plasmodium* sporozoites. TSR domains can be modified with an O-linked fucose by the O-fucosyltransferase POFUT2 [55], and this fucose can be further modified by the enzyme β-1,3-glucosyltransferase to produce a β1,3-linked disaccharide [56–57]. POFUT2 is encoded by a highly conserved *Plasmodium* gene (PF3D7_0909200). There is no annotated *P*. *falciparum* β-1,3-glucosyltransferase; however, BLAST analysis with the human enzyme revealed a protein with 31% identity and 50% similarity. This protein, parasite-infected erythrocyte surface protein or PIESP1 [74] (PF3D7_0310400), is predicted to have glycosyltransferase activity due to the presence of a domain with similarity to Fringe, a beta1,3-N-acetylglucosaminyltransferase that glycosylates O-linked fucose in epidermal growth factor-like domains [57]. Both POFUT2 and PIESP1 are expressed in *P*. *falciparum* salivary gland sporozoites [18]. There is no annotated C-mannosyltrasferase in *Plasmodium*, but BLAST analysis of a recently characterized *C*. *elegans* protein Dpy-19, which adds C-mannose to TSR domains [75], revealed an uncharacterized *P*. *falciparum* membrane protein (PF3D7_0806200) with 26% identity and 47% similarity, including a Dpy-19-like domain annotated by InterPro [76]. This potential glycosyltransferase is also expressed in *P*. *falciparum* salivary gland sporozoites [18].

Structural studies suggest that fucosylation and mannosylation in apicomplexans will make important contributions to antigenic determinants. Crystal structures show that these glycans are well-defined in electron density, and thus have structurally constrained orientations [60, 62]. For example, the glucosylfucose disaccharide is stabilized by its interactions with the disulfide bond, which it shields from solvent [60]. The models of the TSRs of CSP and TRAP based upon crystal structures (Fig 4) suggest that the carbohydrates project prominently above the protein surface, yet do not project nearly as far as, and are unlikely to be nearly as flexible as, N-linked glycans. Fucosylated and mannosylated amino acids may be viewed as surrogate amino acids [77], so the combinatorial protein and carbohydrate determinants create a well-defined recognition surface. For example, Delta-like ligands are modified by an essentially identical glucosylfucose disaccharide, and the carbohydrate is central in the ligand-binding site for Notch [77]. Vertebrate TSR domains contain identical glycans as those we have found in *Plasmodium*; however, the surrounding amino acid residues in parasite TSR domains create unique combinatorial epitopes. Notably, previous studies of naturally-acquired T cell immunity to *P*. *falciparum* CSP have mapped epitopes, including those that correlate with protection, to peptides N-terminal and C-terminal to the fucosylated threonine residue [78–79].

In conclusion, we have employed mass spectrometry-based proteomics to identify putatively surface-exposed proteins of *P*. *falciparum* salivary gland sporozoites. Because the goal of this work was to identify novel antigens for vaccine development efforts, we have combined experimental and theoretical evidence to assign the identified proteins to priority tiers in order to facilitate selection of high-impact targets for follow-up studies. Our investigation included sporozoites treated with molecular mimics of the host environments that sporozoites encounter, providing support that targets of interest are accessible to antibodies throughout the sporozoite’s journey to the liver. Among the more compelling results was the discovery that components of the inner membrane complex appear to be surface-exposed in sporozoites, meaning that this class of proteins may be considered when selecting antigens for vaccine design. Finally, the use of mass spectrometry has enabled us to provide the first direct evidence that the sporozoite surface proteins CSP and TRAP are post-translationally modified with sugars. This information is of critical importance to vaccine design as glycosylation significantly alters recognition epitopes. Taken together, the results that we present here provide significant new information that will aid the development of effective antibody-based therapeutics.

## Materials and Methods

### Ethics Statement

All procedures involving vertebrate animals were conducted in strict accordance with the recommendations in the Guide for the Care and Use of Laboratory Animals of the National Institutes of Health with approved OLAW animal welfare assurance (A3640-01) and Institutional Animal Care and Use Committee (IACUC) protocols (SK-02).

### *Plasmodium falciparum* Culturing and Transmission to Mosquitoes

Standard procedures were used for the culture and transmission of *P*. *falciparum* (NF54 strain) parasites. Briefly, asexual cultures were maintained *in vitro* through infections of washed, type O+ erythrocytes grown in RPMI 1640 supplemented with 50 μM hypoxanthine, 25 mM HEPES, 2 mM L-glutamine, and 10% v/v A+ or O+ human serum in a gas mixture consisting of 5% CO_2_, 5% O_2_, and 90% N_2_. Gametocyte cultures were initiated at 5% hematocrit and 1% parasitemia and were maintained for up to 17 days with daily media changes to promote sexual development. Adult female *An*. *stephensi* mosquitoes (3 to 7 days post-emergence) were collected into mesh-topped, wax-lined pots and were allowed to feed through a membrane feeding apparatus for up to 20 min upon gametocyte cultures supplemented to 40% hematocrit containing fresh A+ or O+ human serum and O+ erythrocytes. Infected mosquitoes were maintained for 14 to 19 days at 27°C and 75% humidity and were provided with an 8% or 20% w/v dextrose solution and 0.05% w/v p-aminobenzoic acid (PABA) in water.

### Sporozoite Isolation, Purification, Viability Assessment, and Surface Labeling

Salivary glands from *P*. *falciparum*-infected mosquitoes were harvested by microdissection and homogenized by grinding. Sporozoite preparations were cleaned with two rounds of purification on an Accudenz discontinuous gradient as previously described [19], washed once with 1 × PBS and resuspended in 630 μL of cold 1 × PBS (pH 8.0 at room temperature (RT)). Total sporozoite numbers were counted on a hemocytometer. Between 4 × 10^6^ and 2 × 10^7^ purified sporozoites were used for each biological replicate. For each experiment, sporozoite viability was assessed by incubating 30 μL of this preparation with 3 μL of 100 μg/mL propidium iodide (PI) for 10 min at RT, followed by a wash in 970 μL 1 × DMEM; sporozoites were pelleted in a microcentrifuge (16,000 × *g* 4 min at 4°C) and then resuspended in 2 μL 1 × DMEM and PI-stained sporozoites were counted by fluorescence microscopy. Typically, about 10% of sporozoites took up the dye.

The remaining 600 μL of sporozoites were used for surface biotinylation. Sporozoites were either left untreated or treated with bovine serum albumin (4% w/v final concentration, Sigma Cat# A7906) at 37°C for 30 min or heparin (0.5 μg/mL final concentration, Sigma Cat# H5515) at 37°C for 10 min. Following treatment, sporozoites were pelleted and washed three times in 1 × PBS pH 7.4. The following steps were performed at 4°C. Surface-exposed proteins on sporozoites were biotinylated by incubating in 1.4 mM EZ-Link Sulfo-NHS-SS-Biotin (Thermo Scientific, Cat# 21331) final concentration for 1 h. The sulfonated-NHS ester is water soluble and cannot penetrate intact cell membranes [20]. The biotinylation reaction was quenched by adding glycine (100 mM final concentration) for 5 min, and sporozoites were then washed twice with 0.5 mL of 100 mM glycine in 1 × PBS pH 8.0. Sporozoites were then resuspended in 100 μL of lysis buffer (0.4% w/v sodium dodecyl sulfate (SDS), 4 M urea, 20 mM Tris-HCl pH 8.0) containing 1 × protease inhibitors (Roche cOmplete protease inhibitor, Cat# 04693159001) for 30 min on a rotating mixer. Lysate was spun at 16,000 × *g* for 25 min and the supernatant was transferred to a new 2-mL tube and diluted tenfold with 1 × PBS (pH 7.4 at RT). One hundred μL of Dynabeads MyOne Streptavidin T1 (Life Technologies, Cat# 65601) were washed three times in 1 × PBS pH 7.4 and incubated with the sporozoite lysate and mixed by end-over-end rotation overnight at 4 ˚C. The following wash steps were performed at RT: Dynabeads were washed sequentially using one of the following three protocols (see S1 Table for sample / wash protocol assignments). Protocol 1: 1) 0.1% w/v SDS in distilled water, 400 mM urea, 150 mM NaCl, 50 mM Tris-HCl pH 8.0; 2) 0.1% w/v SDS in distilled water, 500 mM NaCl, 50 mM Tris-HCl pH 8.0; 3) 0.1% w/v SDS in distilled water, 50 mM Tris-HCl pH 8.0. Protocol 2: 1) 2% w/v SDS in distilled water; 2) 1% v/v Triton X-100, 500 mM NaCl, 1 mM EDTA and 50 mM Hepes pH 7.5; 3) 50 mM Tris pH 7.4 and 50 mM NaCl. Protocol 3: 1) 2% w/v SDS in distilled water; 2) 6 M urea, 2% w/v SDS, 1 M NaCl, 50 mM Tris pH 7.4; 3) 0.2% w/v SDS, 4 M urea, 200 mM NaCl, 1 mM EDTA and 50 mM Tris pH 7.4; 4) 0.2% w/v SDS, 50 mM NaCl and 50 mM Tris pH 7.4. Bound proteins were eluted with 40 μL 2 × sample buffer (50 mM Tris pH 6.8, 5% w/v SDS, 5% v/v glycerol, 0.16% w/v bromophenol blue) to which DTT was added (final concentration of 50 mM) just prior to heating the tube to 70 ˚C for 7 min. The eluted fraction was transferred to a new tube, snap frozen in liquid nitrogen and stored at -80 ˚C until separated by SDS-PAGE.

### Production and Transmission of *Plasmodium yoelii* Transgenic Parasites

*P*. *yoelii* parasites, 17XNL strain, were maintained in six-to-eight week old female Swiss Webster (SW) mice from Harlan (Indianapolis, IN). Transgenic parasites for PY17X_0823700 (putative sugar transporter) and PY17X_1108700 (p38) were created by double-crossover and single-crossover recombination, respectively, to append a C-terminal 3 × HA tag to each protein by electroporating linearized derivatives of the pDEF suite of vectors with the Amaxa Nucleofector 2b system as previously described [80]. Confirmation of the recombination events was achieved by genotyping PCR across both targeting sequence regions as previously described [81]. Transmission of *P*. *yoelii* parasites to *An*. *stephensi* mosquitoes was accomplished by direct feeding on anesthetized, infected mice. Infected mosquitoes were maintained at 24 ˚C and 70% humidity for 14 days.

### Indirect Immunofluorescence Assay Validation of Surface-Exposed and Secreted Proteins

Detection of the expression and localization of proteins of interest by indirect immunofluorescence assay (IFA) was performed by fluorescence microscopy as previously described [81–82]. *P*. *yoelii* wildtype or epitope-tagged sporozoites and liver stage parasites were collected at the indicated time points and were stained with primary antibodies specific to PyCSP (mAb Clone 2F6 [83]), PyTRAP (mAb Clone F3B5), PyUIS4 (rabbit polyclonal antisera [84]), HA epitope tag (rabbit polyclonal antisera, SCBT Cat#Y-11 or mAb Clone 12CA5, Roche Cat#11583816001). Alexa Fluor 488-labeled secondary antibodies against mouse or rabbit IgG were used to detect the proteins of interest, and then sporozoites were stained with 4’,6’-diamidino-2-phenylindole (DAPI) to visualize nucleic acid. Slides were mounted with VectaShield antifade reagent (Vector Laboratory) and images were acquired at 100× using an Olympus IX70 DeltaVision microscope using the softWoRx software package.

### Indirect Immunofluorescence Assay of Inner Membrane Complex Proteins

*P*. *falciparum* salivary gland sporozoites were isolated from *An*. *stephensi* mosquitoes 14 to 17 days post blood feeding. Sporozoites were diluted in cold RPMI 1640 containing 1% w/v BSA and spun onto poly-L-lysine coated coverslips at 300 x g for 4 min at 4°C. To allow sporozoites to glide, samples were incubated at 37°C for 20 min. Following this, samples were taken to the 4°C cold room and fixed in 4% v/v paraformaldehyde in PBS for 10 min. Samples were then washed and incubated in DMEM containing 1% w/v BSA and 5% w/v goat serum for 45 min at RT, labeled with rabbit polyclonal anti-PyMTIP antibody 1:400 [50] or rabbit polyclonal anti-PfGAP45 1:200 (kind gift from Anthony A. Holder, NIMR [85]) for 45 mins at RT then washed again and labeled with Alexa Fluor 488-conjugated goat anti-rabbit IgG for 45 mins at RT before staining with Hoechst 33342 DNA stain for 5 mins. For permeabilization, 0.1% v/v Triton X-100 was included in the blocking and incubation buffers. Live samples were not immediately fixed after transfer to the cold room but instead incubated in DMEM containing 1% w/v BSA and 5% v/v goat serum for 1 h, labeled with anti-PyMTIP antibody 1:400 [50] or anti-PfGAP45 1:200 [85] for 2 h, then washed and fixed in 4% v/v paraformaldehyde in PBS for 2 h. Samples were washed and again incubated in DMEM containing 1% w/v BSA and 5% w/v goat serum for 1 h, and labeled with Alexa Fluor 488-conjugated goat anti-rabbit IgG and Hoechst 33342 DNA stain. Images were acquired on a Zeiss AxioObserver with LSM700 confocal module with the Zeiss ZEN software package or a Nikon Eclipse E600 fluorescence microscope using Nikon Elements software. The proportion of sporozoites displaying strong patches of staining was determined by manual counting of two hundred parasites.

### 1D SDS-PAGE Fractionation

Proteins eluted from magnetic dynabeads were electrophoresed through a 4–20% w/v SDS-polyacrylamide gel at 180 V at 22 ˚C. Gels were post-stained with Imperial Stain (Thermo Scientific) and destained in Milli-Q Water (Millipore, USA). Each gel lane was cut into four to nine fractions (S1 Table) for in-gel tryptic digestion of proteins. Each gel fraction was cut into small pieces (~1 mm) and placed into wells in a 96-well PCR plate. Unless indicated otherwise, all wash and incubation steps were performed at 37°C while agitating the plate at 700 rpm. Gel pieces were destained with three washes of 50 μL of 50 mM ammonium bicarbonate (ABC) in 50% v/v acetonitrile (ACN) for 10 min. The gel pieces were dehydrated with 2 washes of 50 μL of ACN for five min and allowed to dry. The gel pieces were rehydrated with 50 μL of 10 mM dithiothreitol in 100 mM ABC and incubated for 30 min. The unabsorbed reducing solution was discarded and 50 μL of 50 mM iodoacetamide in 100 mM ABC was added and incubated for 20 min. This alkylating solution was discarded and the gel pieces were then washed three times with 50 mM ABC in 50% acetonitrile and dehydrated with three washes of ACN as above. The dried gel pieces were rehydrated with 50 μL of 6.25 ng/μL sequencing grade trypsin (Promega) in 50 mM ABC. Additional 50 mM ABC was added to each well as needed to ensure that the gel pieces were submerged in solution. The plate was incubated overnight without agitation in an oven at 37°C. Tryptic peptides were collected with three extractions: 50 μL of 2% v/v ACN/1% v/v formic acid for 30 min, 50 μL of ACN for 30 min, and 50 μL of 2% v/v ACN/1% v/v formic acid for 30 min. The extracted peptide solutions were combined and evaporated to dryness in a rotary vacuum and reconstituted in 20 μL of 2% v/v ACN/0.2% v/v trifluoroacetic acid.

### Liquid Chromatography-Mass Spectrometry

Nanoflow liquid chromatography (nanoLC) was performed using an Agilent 1100 nano pump with electronically controlled split flow. Peptides were separated on a column with an integrated fritted tip (360 μm outer diameter (O.D.), 75 μm inner diameter (I.D.), 15 μm I.D. tip; New Objective) packed in-house with a 20 cm bed of C18 (Dr. Maisch ReproSil-Pur C18-AQ, 120 Å, 3 μm; Ammerbuch-Entringen, Germany). Prior to each run, sample was loaded onto a trap column consisting of a fritted capillary (360 μm O.D., 150 μm I.D.) packed with a 1 cm bed of the same stationary phase and washed with loading buffer (2% v/v ACN/0.2% v/v trifluoroacetic acid in water). The trap was then placed in-line with the separation column for the separation gradient. The LC mobile phases consisted of 0.1% v/v formic acid in water (solvent A) and 0.1% v/v formic acid in ACN (solvent B). The separation gradient was 5% B to 35% B over 60 min or 90 min, followed by a 10 min ramp to 80% B, a 10 min wash at 80% B, and a re-equilibration step at 5% B. The gradient flow rate was 500 nL/min. Tandem mass spectrometry (MS/MS) was performed with an LTQ Velos Pro-Orbitrap Elite (Thermo Fisher Scientific). Precursor MS1 scans over the range of 400–1600 *m/z* were collected in the Orbitrap with a nominal resolving power of 60,000 at 400 *m/z*. Data-dependent acquisition was employed to select the top 20 doubly- or triply-charged precursors for collision-induced dissociation (CID) and analysis in the ion-trap. Dynamic exclusion was employed with an exclusion list of up to 500 precursors. Precursors were excluded with a +/- 5 ppm tolerance for 30 sec after a single observation or after the precursor level was observed a single time at an intensity below twice the signal-to-noise, whichever came first. Precursors were isolated with a 2.0 *m/z* window and fragmented by CID for 10 ms at a normalized collision energy of 35%. Two nanoLC-MS technical replicates were performed for each fraction, with roughly half the available sample injected for each replicate.

### Peak List Generation

The mass spectrometry data generated for this manuscript, along with the search parameters, analysis parameters and protein databases can be downloaded from PeptideAtlas (www.peptideatlas.org) using the identifier PASS00729. Raw mass spectrometer output files were converted to.mZML format using MSConvert version 3.0.5533 [86] and searched with X!Tandem [87] version 2013.06.15.1 JACKHAMMER and Comet version 2013.2 rev.2 [88]. Spectra were searched against a protein sequence database comprised of the following: *P*. *falciparum* 3D7 (version 10.0, www.plasmodb.org); *A*. *gambiae* (version 3.7, www.vectorbase.org); a modified version of the common Repository of Adventitious Proteins (version 2012.01.01, www.thegpm.org/cRAP,) with the Sigma Universal Standard Proteins removed; the peptides human angiotensin II and [Glu-1] fibrinopeptide B (common MS calibration peptides); the antibody against circumsporozoite protein [89]; and streptavidin (Uniprot ID P22629). Gene annotations of identified *P*. *falciparum* proteins were updated with PlasmoDB PF3D7 version 26. Decoys with randomized sequence were generated using Mimic (www.kaell.org). A wide precursor mass tolerance of 20 ppm was used to improve the performance of the accurate mass binning tool available in PeptideProphet [90]. Fragment ions were searched with a monoisotopic mass error of 0.4 Da in X!Tandem. Fragment ion bins in Comet were set to a tolerance of 1.0005 *m/z* and a monoisotopic mass offset of 0.4 *m/z*. Semi-tryptic peptides and up to 2 missed cleavages were allowed. The search parameters included a static modification of +57.021464 Da at C for carbamidomethylation by iodoacetamide and potential modifications of +15.994915 Da for oxidation at M and +145.019749 Da at K for labeling. (The biotin label contained a disulfide bond which was cleaved and alkylated in the course of sample preparation.) Additionally, X!Tandem automatically searched for potential modifications of -17.026549 Da for deamidation at N-terminal Q and -18.010565 Da for loss of water at N-terminal E from formation of pyro-Glu, as well as -17.026549 Da at N-terminal carbamidomethylated C for deamidation from formation of S-carbamoylmethylcysteine. MS/MS data were analyzed using the Trans-Proteomic Pipeline [91] version 4.7 POLAR VORTEX rev 0. Peptide spectrum matches (PSM) generated by each search engine were analyzed separately with PeptideProphet in order to assess spectral matching quality at the spectrum level, and then the analyses were combined in iProphet with the number of sibling peptides (NSP) model disabled [92]. Protein identifications were inferred with ProteinProphet [93]. In the case that multiple proteins were inferred at equal confidence by a set of peptides, the inference was counted as a single identification and all relevant protein IDs were listed. Only proteins with ProteinProphet probabilities corresponding to a model-estimated false discovery rate (FDR) less than 1.0% were reported.

### Assessing Protein Enrichment

Label-free proteomics methods based on spectral counts (SpC) were used to identify proteins that were significantly more abundant in labeled samples compared to unlabeled controls. A total of 15 biological replicates were analyzed: six untreated, three BSA-treated, three heparin-treated, and three unlabeled controls (S1 Table). The six untreated replicates consisted of two sub-groups (three replicates each) representing two different labs collecting and preparing the samples (S1 Table). For quantitative measurement of protein enrichment by biotinylation, each group of biological triplicates of labeled samples was compared separately against the three control replicates. The SpC for a given protein in a given biological replicate was taken as the number of PSM used by ProteinProphet to make the protein inference. All SpC values were increased by one in order to give all proteins non-zero SpC values for log-transformation [94]. The spectral abundance factor (SAF) for a given protein was calculated as the quotient of the SpC and the protein's length and natural log-transformed to ln(SAF) [24]. While it is common practice to normalize log-transformed SpC or SAF values in order to minimize technical variance, these transformations are based on the assumption that the majority of protein abundances remain unchanged between the compared samples [95]. These conditions were not met in the experiments described here because the unlabeled controls were expected (and observed) to consist of much lower total protein than the labeled samples. Accordingly, we did not normalize the ln(SAF) values. The ln(SAF) values for each protein were compared between labeled and unlabeled samples by a two-tailed, two sample homoscedastic t-test in Microsoft Excel. The FDR associated with multiple hypothesis testing was assessed by the Benjamini-Hochberg method, and p-values corresponding to an FDR less than 10% were considered significantly enriched. Enrichment relative to unlabeled controls was also quantitatively assessed by the program SAINT v2.5.0 [23], which was designed to quantify enrichment in affinity purification experiments using SpC. The following pertinent parameters were used: lowMode = 0, minFold = 1, normalize = 0. Proteins with SAINT probabilities corresponding to a Bayesian FDR less than 10% as calculated by SAINT were considered significantly enriched. For some proteins, labeling with the biotin tag could be directly observed from the mass spectra. A protein was considered to have spectral evidence for labeling if a component peptide displaying the addition of the biotin tag was identified from at least one high-quality spectrum in the same biological replicate in which the protein was inferred by ProteinProphet. For each set of PSMs generated from the Comet or the X!Tandem search of the data from a given biological replicate, the prevalence of PSMs matching decoys among the putatively-labeled spectra was determined and used to calculate a FDR. Only labeled PSMs with PeptideProphet probabilities corresponding to a decoy-estimated FDR less than 1% were counted.

### Prioritization of Identified Proteins

The primary sequences of enriched proteins were analyzed using established tools for predicting the presence of surface protein characteristics. Transmembrane (TM) domains and signal peptide predictions were taken from PlasmoDB.org (*P*. *falciparum* 3D7 version 13) combined with Signal IP version 4.0 (http://www.cbs.dtu.dk/services/SignalP/) and TMHMM version 2.0 (http://www.cbs.dtu.dk/services/ TMHMM-2.0). Glycosylphosphatidylinositol (GPI) anchors were predicted using GPI-SOM (http://gpi.unibe.ch/). For high priority targets, these results were also manually validated based on published literature. All *Plasmodium* proteins identified in each sporozoite treatment condition (untreated, BSA-treated, or heparin-treated), were assigned to tiers in order to prioritize them for follow-up studies. The top tiers consisted of proteins with predicted surface protein characteristics (i.e. signal peptide, TM domain, and/or GPI anchor) and were ranked as follows, in order of decreasing priority: 1) proteins significantly enriched compared to unlabeled controls (as assessed by SAINT) that exhibited spectral evidence for labeling; 2) proteins that were significantly enriched but did not have evidence for labeling or were labeled but not enriched; 3) proteins that were neither enriched nor labeled. Tiers 4, 5 and 6 had the same criteria as tiers 1, 2 and 3, respectively, but were assigned to proteins without predicted surface protein characteristics. Tiers 1 and 2 were considered most likely to be truly surface-exposed in salivary gland sporozoites.

## Supporting Information

S1 Fig Genotyping PCR of transgenic parasites bearing 3 × HA tags on p38 and PY17X_0823700 (PY05332). Gene annotations and genotyping PCR results for the creation of transgenic parasites in which the P38 (A, single crossover) and PY17X_0823700 (B, double crossover) genes are altered to encode a C-terminal 3xHA epitope tag. Left panels of A and B: The recombination strategies are shown and locations of the genotyping PCR products are indicated by lines labeled test 1, test 2 and wt. Right panels of A and B: PCR verification using genomic DNA from transgenic (top) or wild-type (bottom) parasites indicates the presence of the desired transgenic parasites in these populations.
(TIF)

S2 Fig The inner membrane complex proteins MTIP and GAP45 are surface-exposed on live *P*. *falciparum* sporozoites. *P*. *falciparum* sporozoites were allowed to glide on coverslips for 20 min and then moved into the cold room where they were stained for MTIP or GAP45 prior to fixation with 4% v/v paraformaldehyde and detection with Alexa Fluor 488-conjugated secondary antibodies. Strong patches of staining at the ends or middle of sporozoites was observed in a subset of parasites: 38% of those stained for MTIP and 13% of those stained for GAP45. Scale bar is 5 microns.
(TIF)

S1 Table Sample details of biological replicates. (XLSX)

S2 Table *P*. *falciparum* proteins identified from surface labeling of untreated salivary gland sporozoites. (XLSX)

S3 Table *P*. *falciparum* proteins identified from unlabeled salivary gland sporozoites. (XLSX)

S4 Table *P*. *falciparum* proteins identified from surface labeling of BSA-treated salivary gland sporozoites. (XLSX)

S5 Table *P*. *falciparum* proteins identified from surface labeling of heparin-treated salivary gland sporozoites. (XLSX)

S6 Table *P*. *falciparum* proteins with spectral evidence for incorporation of biotin tag. (XLSX)

S7 Table Compiled spectral abundance of all *P*. *falciparum* proteins identified from salivary gland sporozoites. (XLSX)

S8 Table Oligonucleotides used in this study for the creation and genotyping of transgenic *P*. *yoelii* parasites. (XLSX)

S9 Table *P*. *falciparum* proteins identified from surface labeling of salivary gland sporozoites in previously-reported data. (XLSX)
