# Supplementary figures and images for: Interrogating the Plasmodium Sporozoite Surface: Identification of Surface-Exposed Proteins and Demonstration of Glycosylation on CSP and TRAP by Mass Spectrometry-Based Proteomics
Source: PLoS Pathog. 2016 Apr 29;12(4):e1005606. doi: 10.1371/journal.ppat.1005606 (PMC4851412; doi:10.1371/journal.ppat.1005606)

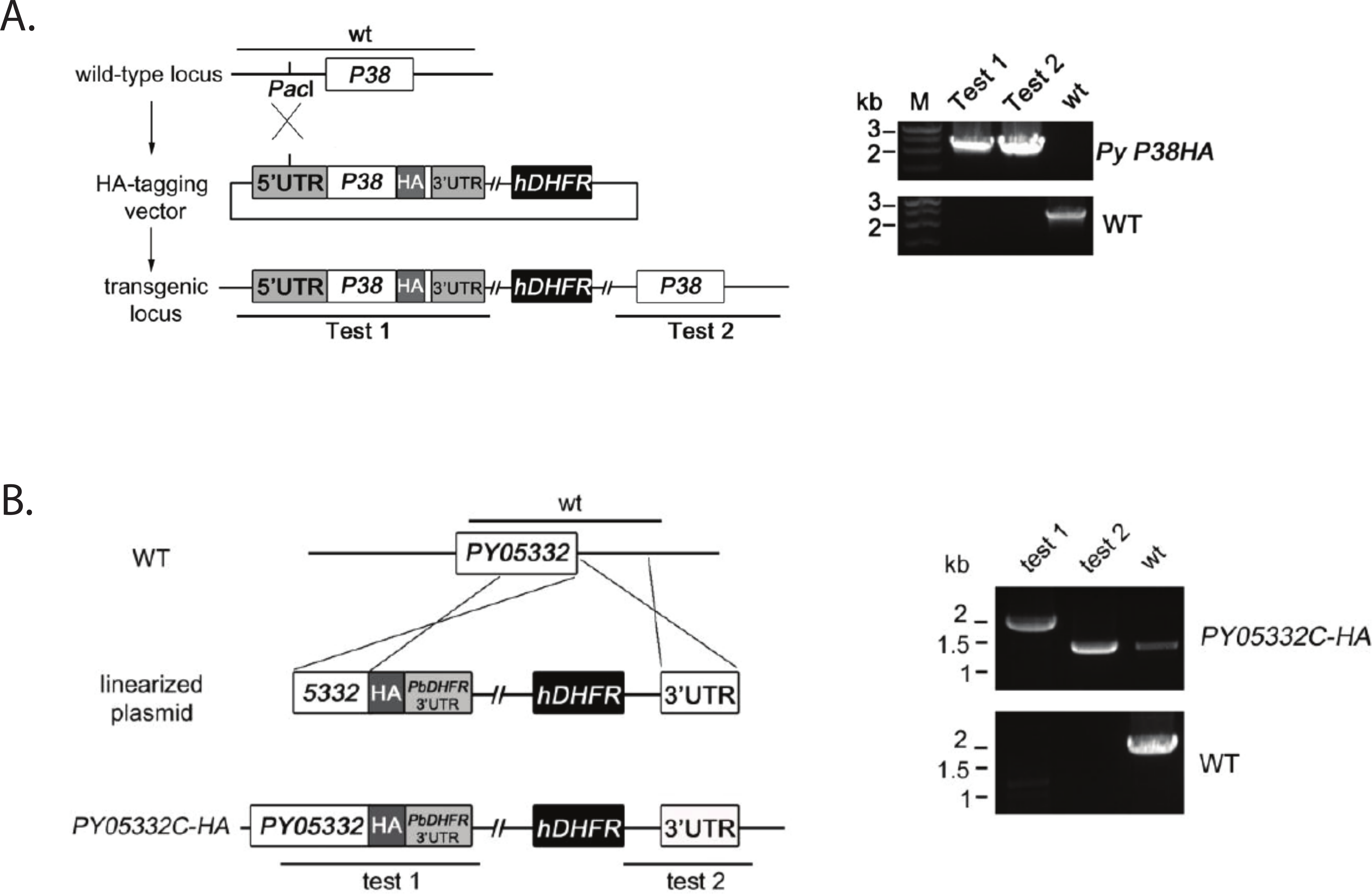

Supplement: S1 Fig — Gene annotations and genotyping PCR results for the creation of transgenic parasites in which the P38 (A, single crossover) and PY17X_0823700 (B, double crossover) genes are altered to encode a C-terminal 3xHA epitope tag. Left panels of A and B: The recombination strategies are shown and locations of the genotyping PCR products are indicated by lines labeled test 1, test 2 and wt. Right panels of A and B: PCR verification using genomic DNA from transgenic (top) or wild-type (bottom) parasites indicates the presence of the desired transgenic parasites in these populations. (TIF) [file ppat.1005606.s001.tif]

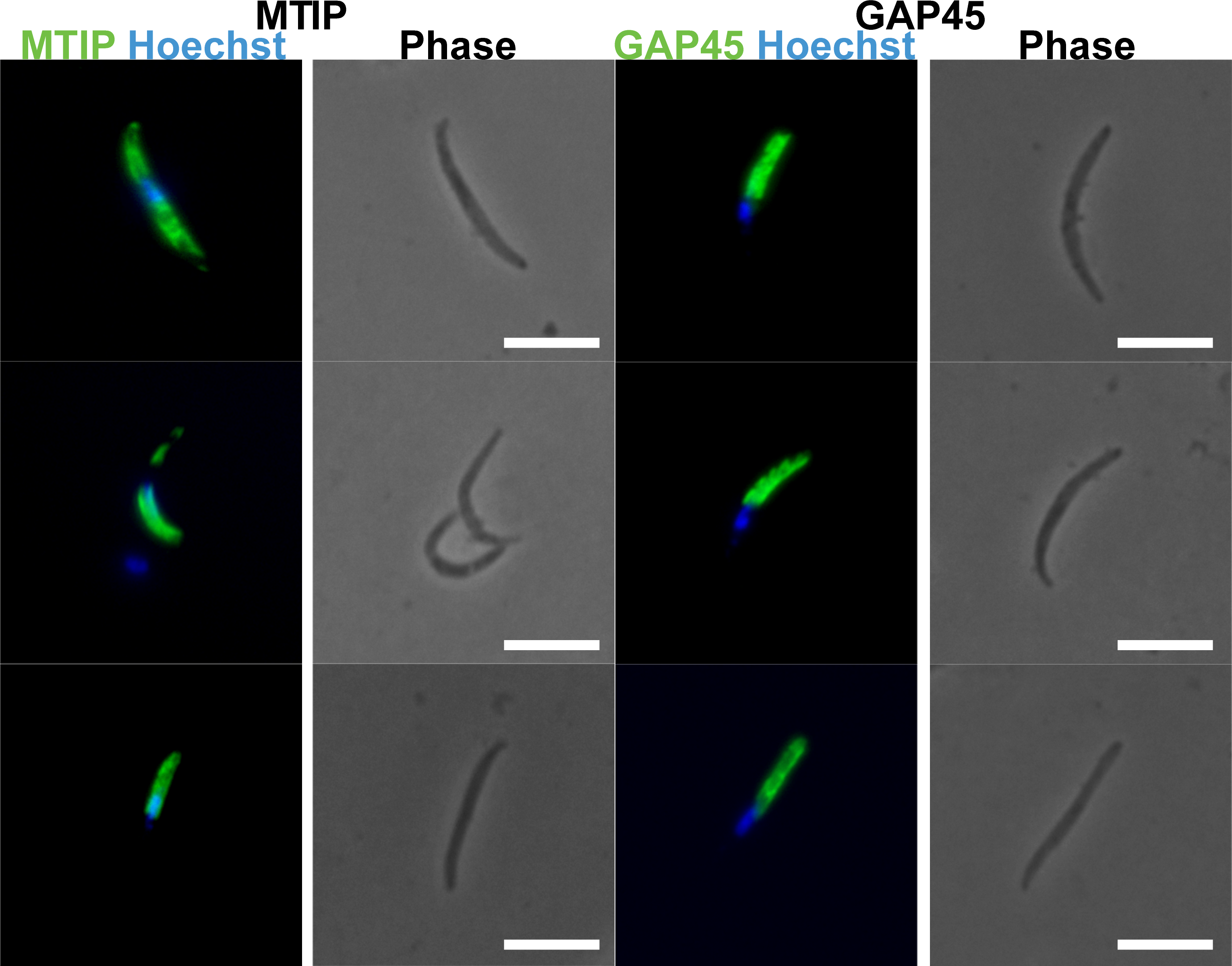

Supplement: S2 Fig — P. falciparum sporozoites were allowed to glide on coverslips for 20 min and then moved into the cold room where they were stained for MTIP or GAP45 prior to fixation with 4% v/v paraformaldehyde and detection with Alexa Fluor 488-conjugated secondary antibodies. Strong patches of staining at the ends or middle of sporozoites was observed in a subset of parasites: 38% of those stained for MTIP and 13% of those stained for GAP45. Scale bar is 5 microns. (TIF) [file ppat.1005606.s002.tif]
